# Lipid transfer proteins: classification, nomenclature, structure, and function

**DOI:** 10.1007/s00425-016-2585-4

**Published:** 2016-08-25

**Authors:** Tiina A. Salminen, Kristina Blomqvist, Johan Edqvist

**Affiliations:** 1 Structural Bioinformatics Laboratory, Biochemistry, Faculty of Science and Engineering, Åbo Akademi University, 20520 Turku, Finland; 2 IFM, Linköping University, 581 83 Linköping, Sweden

**Keywords:** NsLTP, LTP, Cutin, Suberin, Pollen, Protein structure

## Abstract

The non-specific lipid transfer proteins (LTPs) constitute a large protein family found in all land plants. They are small proteins characterized by a tunnel-like hydrophobic cavity, which makes them suitable for binding and transporting various lipids. The LTPs are abundantly expressed in most tissues. In general, they are synthesized with an N-terminal signal peptide that localizes the protein to spaces exterior to the plasma membrane. The in vivo functions of LTPs are still disputed, although evidence has accumulated for a role in the synthesis of lipid barrier polymers, such as cuticular waxes, suberin, and sporopollenin. There are also reports suggesting that LTPs are involved in signaling during pathogen attacks. LTPs are considered as key proteins for the plant’s survival and colonization of land. In this review, we aim to present an overview of the current status of LTP research and also to discuss potential future applications of these proteins. We update the knowledge on 3D structures and lipid binding and review the most recent data from functional investigations, such as from knockout or overexpressing experiments. We also propose and argument for a novel system for the classification and naming of the LTPs.

## Introduction

The non-specific lipid transfer proteins (LTPs) were first discovered approximately 35 years ago. Since then the LTP family has expanded, but it has still kept its secrets unrevealed for plant biologists. The LTPs are found in all land plants, encoded by large gene families, and abundantly expressed in most tissues. Their vast abundance indicates their importance for the survival and reproduction of plants. A search on PubMed in October 2015 with the terms “plant” and “lipid transfer protein” revealed more than 700 papers published dealing with different aspects of the LTPs. A quite large proportion of these reports, approximately 30 %, are focusing on the allergenic properties of the LTPs. There are also many publications that cover different biochemical aspects, such as their structure, ligand binding, and regulation. However, although a quite large number of reports have been published, we still have a rather limited understanding of the basic physiological function of the LTPs. This is probably due to that it has been difficult to find good tools and strategies for conclusive experiments. In recent years though, several papers have appeared that reveal phenotypes after knocking-down, knocking-out or increasing the expression of LTPs. It seems that we are slowly gaining some functional understanding of these proteins. Therefore, it is a good time to review the literature, present the current ideas regarding the biological function, and discuss the future directions for research about the LTPs.

## Features and classification of LTPs

The LTPs are small and soluble, cysteine-rich proteins. Their molecular size is usually below 10 kDa (Kader 1996). They possess four or five α-helices, which are stabilized by four conserved disulfide bridges formed by an eight-Cys motif (8CM) with the general form C-Xn-C-Xn-CC-Xn-CXC-Xn-C-Xn-C. The disulfide bridges promote the folding of the LTP into a very compact structure, which is extremely stable to heat and denaturation agents (Lindorff-Larsen and Winther 2001; Berecz et al. 2010; Edstam et al. 2014). The LTPs are in general synthesized with an N-terminal signal peptide that localizes the protein to the apoplastic space. They are abundant in all investigated land plants, but absent from chlorophyte and charophyte green algae as well as all other organisms (Edstam et al. 2011). The LTPs are encoded by large gene families with more than 50 members in many flowering plants and up to 50 members in bryophytes and ferns (Boutrot et al. 2008; Edstam et al. 2011; Li et al. 2014a; Wei and Zhong 2014). Several LTPs are known to cause plant food allergies in humans. Curiously, these LTP allergies are frequent in Mediterranean countries but rare in Northern Europe. The role of LTPs in allergic reactions is not covered in this review where we focus on the biological function of LTPs in plants. We would rather recommend other reviews for an update on this important and interesting aspect of LTPs (Egger et al. 2010; Salcedo et al. 2007; Van Winkle and Chang 2014).

The LTPs are often simply classified into either of the types LTP1 or LTP2. These types differ by their molecular size as LTP1s have about 90 amino acids and LTP2s have about 70 amino acids (Kalla et al. 1994). A second LTP classification system based on sequence identity has also been introduced (Boutrot et al. 2008). When the LTPs were characterized in early diverging plants, such as mosses and liverworts, the LTPs in those plants could not readily be classified into LTP1 or LTP2 due to the variations in molecular size. Furthermore, the limited sequence conservation made it unsuitable to apply the sequence-based sorting system. Therefore, we introduced a modified and expanded LTP-classification system yielding five major types (LTP1, LTP2, LTPc, LTPd, and LTPg) and four minor types with fewer members (LTPe, LTPf, LTPh, LTPj, and LTPk) (Edstam et al. 2011). This classification system is not based on the molecular size, but rather on the position of a conserved intron, the amino acid sequence identity and the spacing between the Cys residues in the 8CM. The system also considers post-translational modifications, e.g., LTPs with a GPI-anchor belong to LTPg. Since this novel classification system assays several features of the LTPs, it is more robust than previous classification systems (Joly and Matton 2015). We would, therefore, recommend it for future classifications of the LTPs. Although the classification system is novel, the conventional classification of LTP1 and LTP2 types is preserved.

## Distribution and nomenclature

When we applied the novel classification system, we found that non-seed plants have a more limited set of LTP types compared with seed plants. This indicates that novel LTP types have evolved during land plant evolution. LTPd and LTPg are found in all investigated land plants from bryophytes to flowering plants and, therefore, represent the earliest LTPs (Edstam et al. 2011). LTP1 and LTPc are restricted to vascular plants, while LTP2 is further limited to seed plants. Since we entered the era of plant genome sequencing, the complete array of LTP genes has been deduced for several plant species (Table 1). Curiously, the genome-wide search of the moss *Physcomitrella patens* revealed two genes encoding proteins with two connected 8CMs and another gene encoding three 8CMs (Edstam et al. 2011). So far, the multidomain LTPs are uniquely identified in *P. patens*.

**Table 1:** Distribution of LTPs in some selected plant genomes

Plant species	Total LTPs	LTP1	LTP2	LTPc	LTPd	LTPe	LTPf	LTPg	LTPh	LTPj	LTPk	LTPx^g^
*Marchantia polymorpha* ^a,e^	14				8			4				2
*Physcomitrella patens* ^a,f^	40				21			10		7	2	
*Selaginella moellendorffii* ^a,f^	43				19	3	2	12	6			1
*Pinus taeda* ^a,e^	42	9	1	2	12		1	17				
*Oryza sativa* ^a,f^	77	20	13	2	12			27				3
*Oryza sativa* ^b,f^	77	18	13	2	14			27				3
*Zea mays* ^b,f^	51	8	9	2	16			26				2
*Sorghum bicolor* ^b,f^	58	9	7	2	13			24				3
*Arabidopsis thaliana* ^a,f^	79	12	14	3	12	2		34				4
*Arabidopsis thaliana* ^b,f^	78	13	13	2	12	2		29				7
*Arabidopsis thaliana* ^c,f^	79	12	15	3^h^	12^i^	2^j^		31				2^k^
*Brassica napa* ^d,f^	85	19	15	3^h^	21^i^	3^j^		22				2^k^

^a^Edstam et al. (2011)

^b^Wei and Zhong (2014)

^c^Boutrot et al. (2008)

^d^Li et al. (2014a)

^e^Data from cDNA and EST analysis

^f^Data from genome-wide analysis

^g^Proteins that fulfill the criteria for LTP but which not share characteristics with the other LTP types are placed in the column LTPx

^h^Type III in Boutrot et al. (2008) and Li et al. (2014a)

^i^Types IV, V, VI, VIII, and XI in Boutrot et al. (2008) and Li et al. (2014a)

^j^Type IX in Boutrot et al. (2008) and Li et al. (2014a)

^k^NsLTPy in Boutrot et al. (2008) and Li et al. (2014a)

The naming of LTPs has been confusing and without any guidelines or standardization. There are, for instance, several examples where specific LTPs are given different names in separate papers. The lack of a robust naming system has occasionally made it rather difficult, extremely time-consuming and sometimes also frustrating to compare the data from different papers. We would, therefore, encourage the use of a well-defined, simple but informative naming system for the LTPs. The following format is suggested for naming the LTPs: AtLTP1.3, OsLTP2.4, HvLTPc6, PpLTPd5, and TaLTPg7. The first two letters indicate the plant species (At = *Arabidopsis thaliana,* Pp = *Physcomitrella patens* etc.), LTP1, LTP2, LTPc indicate the type, while the last digit (here 3–7) indicates the specific number given to each gene/protein within a certain LTP type. For clarity, we recommend that a punctuation mark is placed between the type specification and gene number in LTP1 and LTP2. For LTPc, LTPd, LTPg, and other LTP types defined with a letter, the punctuation mark is not needed. This naming system was introduced previously for *Marchantia polymorpha*, *P. patens*, *S. moellendorffii*, *Adiantum capillus*-*veneris*, *Pinus taeda*, and Arabidopsis (Edstam et al. 2011, 2013; Joly and Matton 2015) and later used also for maize, *Oryza sativa* (rice) and sorghum (Wei and Zhong 2014). In this review, we also introduce the novel naming system to LTPs from other plants, such as *Hordeum vulgare* (barley), *Triticum aestivum* (wheat), and *Nicotiana tabacum* (tobacco) (Table 2).

**Table 2:** The LTPs described in this review

***Species***	***Current name*** *(This publication)*	***Other names*** *(Miscellaneous publications)*	***UniProt Id***	
*Triticum aestivum* (wheat)	TaLTP1.1	LTP1 (Gincel et al. 1994; Charvolin et al. 1999), TaLtp9.1a (Boutrot et al. 2007); TaLtpIa.1 (Boutrot et al. 2008)	P24296; Q8GZB0	
	TaLTP1.2	TaLtp9.1b (Boutrot et al. 2007); TaLtpIa.2 (Boutrot et al. 2008)	Q5NE27	
	TaLTP1.3	TaLtp9.2b (Boutrot et al. 2007) ; TaLtpIb.1 (Boutrot et al. 2008)	Q5NE28	
	TaLTP1.4	TaLtp9.2c (Boutrot et al. 2007); TaBs116G9 (Sun et al. 2008); TaLtpIb.2 (Boutrot et al. 2008)	Q2PCC2	
	TaLTP1.5	TaLtp9.2d (Boutrot et al. 2007); TaLtpIb.3 (Boutrot et al. 2008)	Q2PCC1	
	TaLTP1.6	TaLtp9.3a (Boutrot et al. 2007); TaLtpIc.1 (Boutrot et al. 2008)	Q5NE30	
	TaLTP1.7	TaLtp9.3b (Boutrot et al. 2007); TaLtpIc.2 (Boutrot et al. 2008)	Q2PCE0	
	TaLTP1.8	TaLtp9.3c (Boutrot et al. 2007); TaLtpIc.3 (Boutrot et al. 2008)	Q2PCD9	
	TaLTP1.9	TaLtp9.3d (Boutrot et al. 2007); TaLtpIc.4 (Boutrot et al. 2008)	Q2PCB9	
	TaLTP1.10	TaLtp9.3e (Boutrot et al. 2007); TaLtpIc.5 (Boutrot et al. 2008)	Q2PCD7	
	TaLTP1.11	TaLtp9.3f (Boutrot et al. 2007); TaLtpIc.6 (Boutrot et al. 2008)	Q5NE33	
	TaLTP1.12	TaLtp9.3g (Boutrot et al. 2007); TaLtpIc.7 (Boutrot et al. 2008)	Q2PCD4	
	TaLTP1.13	TaLt19C10, TaBs112C7 (Gaudet et al. 2003; Sun et al. 2008); TaLtpIb.5 (Boutrot et al. 2008)	Q1KMU9	
	TaLTP1.14	TaLtp9.4a (Boutrot et al. 2007); TaLtpId.1 (Boutrot et al. 2008); Qfhs.ifa-5A (Schweiger et al. 2013)	Q5NE29	
	TaLTP1.15	TaLtp9.4b (Boutrot et al. 2007); TaLtpId.2 (Boutrot et al. 2008)	Q2PCB6	
	TaLTP1.16	TaLTP3 (Jang et al. 2005; Saltzmann et al. 2010; Wang et al. 2010); TaLtp9.4c (Boutrot et al. 2007); TaLtpId.3 (Boutrot et al. 2008)	Q84N29	
	TaLTP1.17	TaLTP1 (Jang et al. 2005); TaLtp9.5a (Boutrot et al. 2007); TaLt710H24 (Sun et al. 2008); TaLtpIb.33 (Boutrot et al. 2008)	Q9FUK0	
	TaLTP1.18	TaLTP2 (Jang et al. 2005); TaLtp9.5b (Boutrot et al. 2007); TaLt709L6 (Sun et al. 2008); TaLtpIb.34 (Boutrot et al. 2008)	Q9ATG4	
	TaLTP1.19	TaLtp9.6a (Boutrot et al. 2007); TaLtpIf.1 (Boutrot et al. 2008)	Q5NE32	
	TaLTP1.20	TaLtp9.7a (Boutrot et al. 2007); TaLtpIg.1 (Boutrot et al. 2008)	Q5NE31	
	TaLTP1.21	TaLtp9.7b (Boutrot et al. 2007); TaLtpIg.2 (Boutrot et al. 2008)	Q2PCD2	
	TaLTP1.22	TaLtp9.7c (Boutrot et al. 2007); TaLtpIg.3 (Boutrot et al. 2008)	Q2PCD1	
	TaLTP1.23	TaLtp9.7d (Boutrot et al. 2007); TaLtpIg.4 (Boutrot et al. 2008); Hfr-LTP (Saltzmann et al. 2010)	Q2PCB7	
	TaLTP1.24	TaLtp9.7e (Boutrot et al. 2007); TaLtpIg.5 (Boutrot et al. 2008)	Q2PCB8	
	TaLTP1.25	TaLTP5 (Zhu et al. 2012)	J9T0L6	
	TaLTP1.26	TaLt10B6 (Gaudet et al. 2003; Sun et al. 2008)	Q1KMV1	
	TaLTP1.27	TaBs108F7 (Sun et al. 2008)	NA	
	TaLTP1.28	TaLt10F9; TaLt10E10 (Gaudet et al. 2003; Sun et al. 2008)	Q1KMV0	
	TaLTP1.29	Ltp 3F1 (Kirubakaran et al. 2008)	A4GU98	
	TaLTP2.1	LTP2 (Douliez et al. 2001; Pons et al. 2003); TaLTP7.1a (Boutrot et al. 2007); TaLtpIIa.1 (Boutrot et al. 2008)	P82900	
	TaLTP2.2	TaLTP7.1b (Boutrot et al. 2007); TaLtpIIa.2 (Boutrot et al. 2008)	Q2PCC3	
	TaLTP2.3	TaLTP7.1c (Boutrot et al. 2007); TaLtpIIa.3 (Boutrot et al. 2008)	Q2PCC7	
	TaLTP2.4	TaLTP7.1e (Boutrot et al. 2007); TaLtpIIa.5 (Boutrot et al. 2008)	Q2PCC5	
	TaLTP2.5	TaLTP7.2a (Boutrot et al. 2007); TaLtpIIb.1 (Boutrot et al. 2008)	Q5NE34	
	TaLTPd1	TaPR60 (Kovalchuk et al. 2009)	B2C4K0	
	TaLTPd2	TaPR61 (Kovalchuk et al. 2012)	H9U3X3	
*Triticum durum* (durum wheat)	TdLTPd1	TdPR60 (Kovalchuk et al. 2009)	C7AE88	
	TdLTPd2	TdPR61 (Kovalchuk et al. 2012)	H9U3X2	
*Hordeum vulgare* (barley)	HvLTP1.1	bLTP (Lerche et al. 1997); ns-LTP_barley_ (Lerche and Poulsen 1998); LTP1 (Lindorff-Larsen et al. 2001)	P07597	
	HvLTP1.2	LTP7a2b (Hollenbach et al. 1997)	Q42848	
*Nicotiana tabacum* (tobacco)	NtLTP1.1	LTP1_1 (Da Silva et al. 2005)	Q42952	
	NtLTP1.2	NtLTP1 (Choi et al. 2012)	Q8LK72	
	NtLTP1.3	NtLTP2 (Choi et al. 2012)	E3W9R1	
	NtLTP1.4	NtLTP3 (Choi et al. 2012)	F2ZAM0	
	NtLTP1.5	NtLTP4 (Choi et al. 2012)	F2ZAM1	
	NtLTP1.6	TobLTP2 (Masuta et al. 1992; Nieuwland et al. 2005)	Q03461	
*Ginkgo biloba* (ginkgo)	GbLTP1.1	Gb-nsLTP1 (Sawano et al. 2008)	A9X6V0	
*Vigna radiata* (mungbean)	VrLTP1.1	Mb nsLTP1 (Lin et al. 2005)	P83434	
	VrLTP1.2	Vrltp1 (Liu and Lin, 2003)	Q6WAT9	
	VrLTP1.3	Vrltp2 (Liu and Lin, 2003)	Q6WAT8	
*Vigna unguiculata* (cowpea)	VuLTP1.1	VULTP (Carvalho et al. 2006)	NA	
*Lillium longiflorum* (lily)	LlLTP1.1	SCA (Park et al. 2000)	Q9SW93	
*Senecia squalidus*	SsLTP1.1	*S. squalidus* GO255151 (Allen et al. 2010)	NA	
*Astragalus sinicus* (Chinese milk vetch)	AsLTP1.1	AsE246 (Lei et al. 2014).	Q07A25	
*Coffea arabica*	CaLTP2.1	CaLTP1a, CaLTP2 (Cotta et al. 2014)	S6FDF9	
	CaLTP2.2	CaLTP1b (Cotta et al. 2014)	S6EPL2	
	CaLTP2.3	CaLTP3b (Cotta et al. 2014)	S6FQL6	
	CaLTP2.4	CaLTP3a (Cotta et al. 2014)	S6DRK0	
*Capsicum annuum* L (chili pepper)	CaLTPc1	CaMF2 (Chen et al. 2011)	F6LQG2	
*Brassica rapa*	BrLTPd1	BraLTP1, Bra011229 (Liu et al. 2014)	M4D425	
*Medicago truncatula*	MsLTPd1	MtN5 (Pii et al. 2009)	O24101	
*Lens culinaris* (lentil)	LcLTP1.1	Lc-LTP1 (Finkina et al. 2007)	A0AT28	
	LcLTP1.2	Lc-LTP2 (Finkina et al. 2007); Len c 3 (Akkerdaas et al. 2012)	A0AT29	
	LcLTP1.3	Lc-LTP3 (Finkina et al. 2007)	A0AT30	
	LcLTP1.4	Lc-LTP4 (Finkina et al. 2007)	A0AT33	
	LcLTP1.5	Lc-LTP5 (Finkina et al. 2007)	A0AT31	
	LcLTP1.6	Lc-LTP6 (Finkina et al. 2007)	A0AT32	
*Prunus persica* (peach)	PpLTP1.1	Pru p 3 (Fernández-Rivas et al. 2003)	Q9LED	
*Corylus avellana* (hazelnut)	CaLTP1.1	Cor a 8 (Offermann et al. 2015)	Q9ATH2	
	Current name (Edstam et al. 2011)			Gene Id
*Arabidopsis thaliana*	AtLTP1.5	LTP1 (Arondel et al. 2000; Chae et al. 2010)	Q42589	At2g38540
	AtLTP1.4	LTP2 (Arondel et al. 2000; Chae et al. 2010)	Q9S7I3	At2g38530
	AtLTP1.12	LTP3 (Arondel et al. 2000; Jung et al. 2003; Chae et al. 2010; Guo et al. 2013b)	Q9LLR7	At5g59320
	AtLTP1.11	LTP4 (Arondel et al. 2000; Jung et al. 2003; Chae et al. 2010)	Q9LLR6	At5g59310
	AtLTP1.8	LTP5 (Arondel et al. 2000; Chae et al. 2010)	Q9XFS7	At3g51600
	AtLTP1.6	LTP6 (Arondel et al. 2000; Chae et al. 2010)	F4IXC6	At3g08770
	AtLTP1.1	LTP7 (Arondel et al. 2000; Chae et al. 2010)	Q9ZUK6	At2g15050
	AtLTP1.3	LTP8 (Arondel et al. 2000; Chae et al. 2010)	Q9ZPW9	At2g18370
	AtLTP1.2	LTP9 (Arondel et al. 2000; Chae et al. 2010)	Q6AWW0	At2g15325
	AtLTP1.10	LTP10 (Arondel et al. 2000; Chae et al. 2010)	Q9LZV9	At5g01870
	AtLTP1.9	LTP11 (Arondel et al. 2000; Chae et al. 2010)	Q2V3C1	AT4G33355
	AtLTP1.7	LTP12 (Arondel et al. 2000; Chae et al. 2010)	Q9SCZ0	At3g51590
	AtLTPd1	DIR1 (Maldonado et al. 2002)	Q8W453	At5g48485
	AtLTPd2	DIR1-like (Champigny et al. 2013)	Q84WQ6; Q9LV65	At5g48490
	AtLTPd9	END1 (Li et al. 2014b)	Q9LQN1	At1g32280
	AtLTPd12	END2 (Li et al. 2014b)	Q9FM83	At5g56480
	Current name (Edstam et al. 2013)			
	AtLTPg1	LTPG1 (Debono et al. 2009; Lee et al. 2009)	Q9C7F7	At1g27950
	AtLTPg2	LTPG2 (Kim et al. 2012)	Q9LZH5	At3g43720
	AtLTPg3		Q9LE56	At1g18280
	AtLTPg4		Q2PE70	At4g08670
	AtLTPg5		Q9LJ86	At3g22600
	AtLTPg6		Q9C896	At1g55260
	AtLTPg23		Q2PE60	At1g36150
	AtLTPg26		Q2PE59	At4g14815
	Current name (Edstam et al. 2013; Wei and Zhong, 2014)			Locus name
*Oryza sativa* (rice)	OsLTP1.18	LTP (Lee et al. 1998)	Q0IQK9	Os12g0115100, LOC_Os12g02320
	OsLTP2.3	LTP-2 (Samuel et al. 2002)	Q10ST8	Os03g0111300, LOC_Os03g02050
	OsLTPd11	OsLTP6 (Liu et al. 2013), OsDIL (Guo et al. 2013a)	Q10A49; Q33B26	Os10g0148000, LOC_Os10g05720
	OsLTPg1		Q8RZK6	Os01g0814100; LOC_Os01g59870
	OsLTPg2		Q10R96	Os03g0167000; LOC_Os03g07100
	OsLTPg24		Q0D9K5	Os06g0711900; LOC_Os06g49770
	OsLTPg25	OsC6 (Zhang et al. 2010)	Q2R222	Os11g0582500, LOC_Os11g37280
*Zea mays* (maize)	ZmLTP1.2	Zm-LTP	O24583	GRMZM2G010868
	ZmLTP1.6	LTP (Gomar et al.^1996^; Shin et al. 1995); Zea m 14 (Pastorello et al. 2000)	P19656	GRMZM2G101958
	ZmLTPd6	BETL9 (Royo et al. 2014)	B6SHX0; C5JA67	GRMZM2G087413
	ZmLTPd14	BETL9like (Royo et al. 2014)	B4FFB8	GRMZM2G091054

## Ligand binding and 3D structure

### Wheat LTP1

**Fig. 1:**
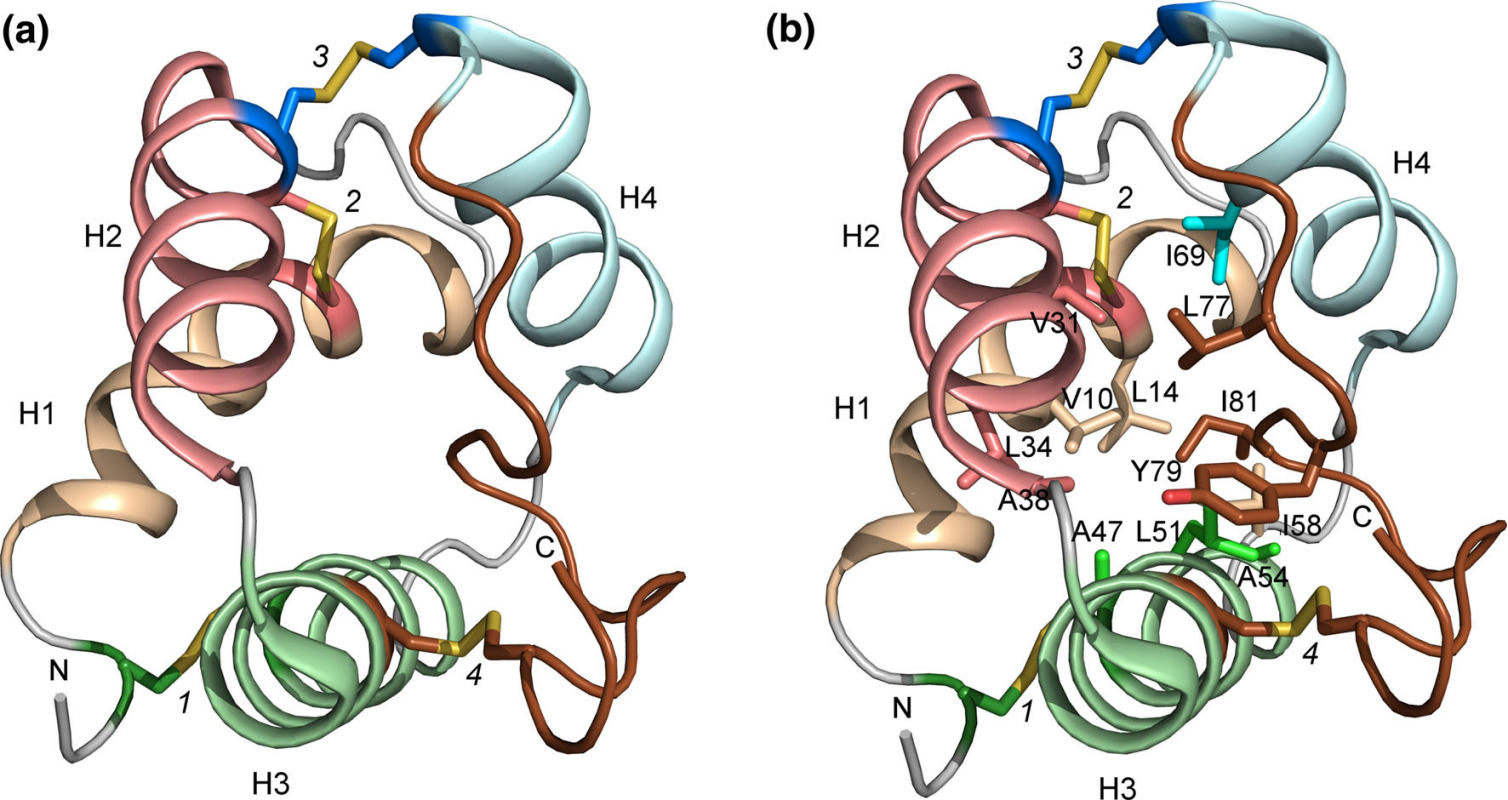
The 3D structure of TaLTP1.1. **a** The four helices in the 3D-fold of TaLTP1.1 are stabilized by four disulfide bridges. The first bridge (*1*; Cys residues shown as *green sticks*) links the N-terminal part (N) to H3 (*green*), the second one (*2*; Cys as *pink sticks*) connects H1 (*wheat*) to H2 (*pink*), the third one (*3*; Cys as *blue sticks*) connects H2 (*pink*) to H4 (*pale cyan*), and the last disulfides bridge (*4*; Cys as *brown sticks*) binds the C-terminal part (C; *brown*) to H3. **b** The internal cavity of TaLTP1.1 is formed by residues from each of the helices. The residues lining the cavity are shown as *sticks* and colored similarly as the helices

The 3D structures of LTPs have been determined using both NMR spectroscopy and X-ray crystallography, either in free, unliganded form or in a complex with ligands (liganded form). The first 3D structure of an LTP was established on the basis of 3D and 2D ^1^H-NMR data of an aqueous solution of TaLTP1.1 purified from wheat seeds (Simorre et al. 1991; Gincel et al. 1994: Protein Data Bank Identification Code (PDB ID) 1GH1). The structure revealed four helices linked together by flexible loops and packed against the unstructured C-terminal part (Fig. 1a), which is stabilized by several hydrogen bonds. The four disulfide bridges formed by the eight Cys in the 8CM stabilize the fold of the protein. Both the N-terminal end of helix 1 (H1) and the C-terminal part are linked to helix 3 (H3) by disulfide bridges (marked 1 and 4 in Fig. 1a), respectively. The position of helix 2 (H2) is stabilized by two disulfide bonds; one of them links the N-terminal part of H2 to the C-terminal part of H1 and the other one links H2 to helix 4 (H4) (bridges 2 and 3 in Fig. 1a). The central hydrophobic cleft is formed by the residues from H1 (Val10 and Leu14), H2 (Val31, Leu34, and Ala38), H3 (Ala47, Leu51, and Ala54), and loop H3–H4 (Ile58), H4 (Ile69), and from the C-terminal part (Leu77, Tyr79, and Ile81) (Fig. 1b).

Glycerophospholipids, such as derivatives of phosphatidylglycerol (PG) or phosphatidylcholine (PC), are important membrane components in most cells. In plants, PG is an important component of the thylakoids, whereas PC makes up a very high proportion of the outer leaflet of the plasma membrane. According to experiments performed with ^1^H NMR and fluorescence spectroscopy, the wheat LTP TaLTP1.1 can fit the PG derivative 1,2-dimyristoyl phosphatidylglycerol (DMPG) in its binding cavity (Sodano et al. 1997). In DMPG, two myristoyl chains are connected to the sn1 and sn2 positions of the PG backbone. In the TaLTP1.1:DMPG complex, both acyl chains are accommodated into the hydrophobic cavity. The volume of the cavity was estimated to be 750 ± 250 Å^3^ when occupied by the two acyl chains. The fold of the LTP was only weakly affected by the insertion of the bulky lipid. The only structural alteration induced by DMPG is seen in the C-terminal part of the structure where the aromatic ring of Tyr79 is directed outwards into the solvent in the TaLTP1.1:DMPG complex, excluding the formation of hydrogen bonds between DMPG and TaLTP1.1.

Experiments assaying the ligand binding of LTPs are often based on competition between labeled lipid analogs and unlabeled fatty acids or lipids. Fluorescent fatty acid analogs, such as anthroyloxy-fatty acids, 1-pyrenedodecanoic acid (P-96), and 2-*p*-toluidinonaphtalene-6-sulfonate (TNS), have been useful tools in the competition assays (Buhot et al. 2004; Zachowski et al. 1998). When the capacity of fatty acids to displace 12-anthroyloxystearate from the cavity of wheat TaLTP1.1 was investigated, an increased number of *cis*-double bonds in the tested C18 fatty acids led to a lower displacement power. A single unsaturation, though, did not affect the affinity of the fatty acid for the protein (Guerbette et al. 1999a).

**Fig. 2:**
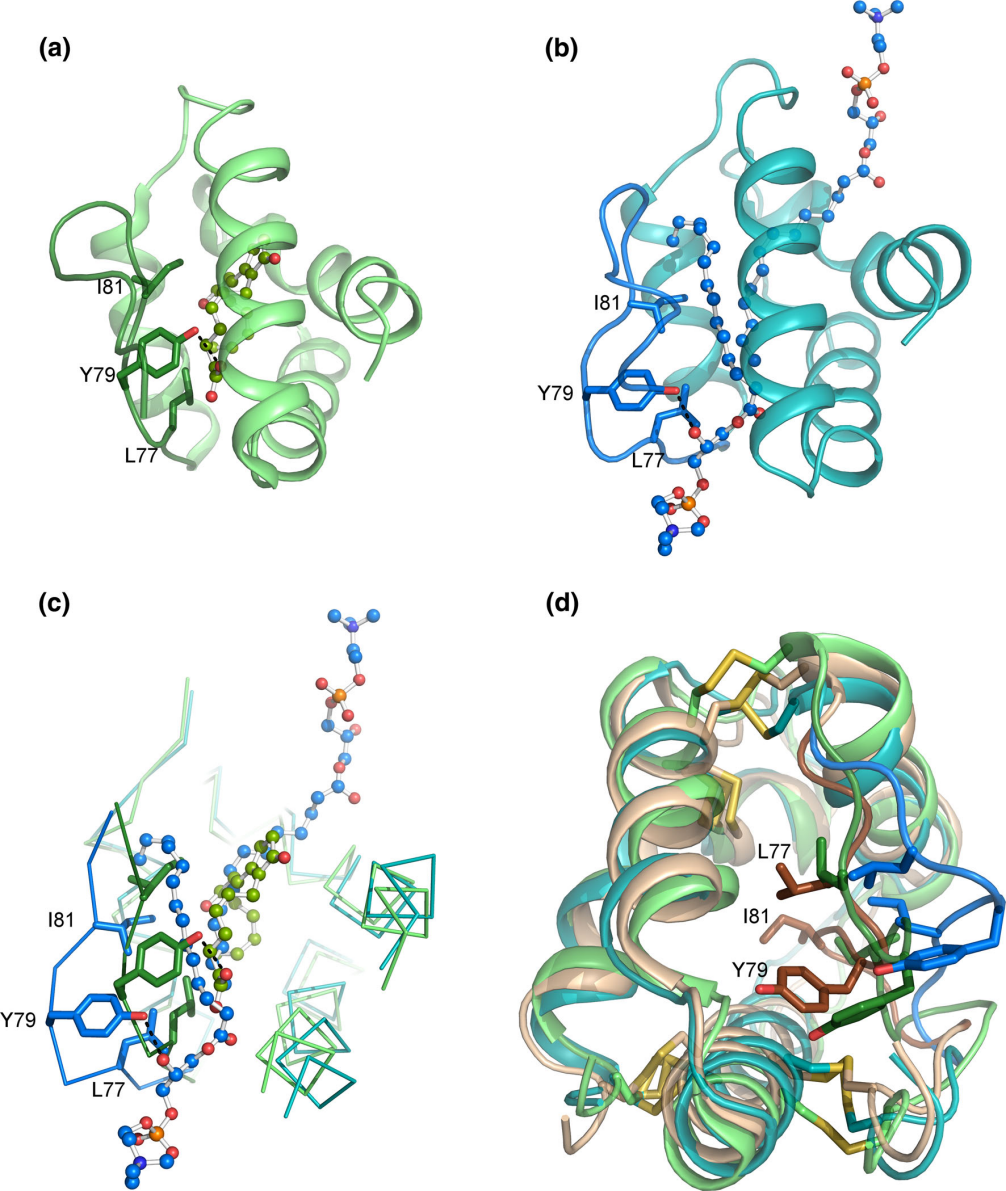
Ligand-binding properties of TaLTP1.1. The hydrogen bonds formed by Tyr79 and the ligands are shown with dashed line. **a**–**c** are in the same orientation. **a** The TaLTP1.1:PGB_2_ complex. **b** The TaLTP1.1:LMPC complex. **c** The structural differences between the PGB_2_ and LMPC complexes. **d** A comparison of unliganded TaLTP1.1 (*wheat*) with the ligand bound forms (*green* and *blue*). The comparison shows clearly that the C-terminal part with residues Leu77, Tyr79, and Ile81 (residues 75–84 in *darker* color) makes major movements depending on the size of the ligand

Many lyso-PC (LPC) or lyso-PG (LPG) derivatives (Table 3) have been used for investigating the ligand binding of LTPs. In all the LPC or LPG derivatives, only one fatty acyl chain is connected to the PC or PG backbone. The 2.1-Å crystal structure of TaLTP1.1:LMPC (Charvolin et al. 1999; PDB ID 2BWO) showed that TaLTP1.1 can accommodate two molecules of LMPC (Fig. 2b; Table 3). The two lipids are positioned head to tail. The aliphatic chains are positioned inside the cavity, while the polar head groups are directed toward the solvent areas, at each end of the tunnel. In site 1, LMPC contacts wheat TaLTP1.1 via hydrophobic interactions and through a hydrogen bond with the side chain hydroxyl of Tyr79, whereas in site 2, LMPC is only involved in a few hydrophobic interactions (Fig. 2b).

**Table 3:**
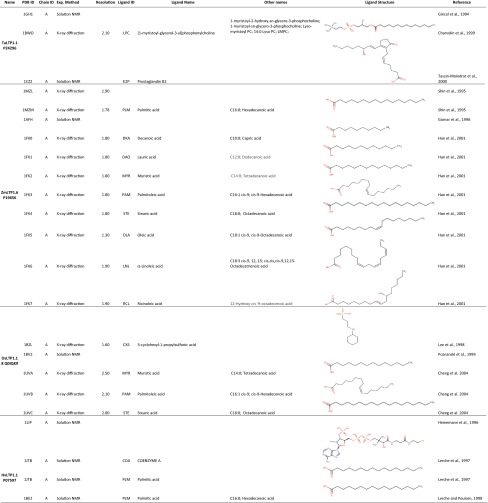

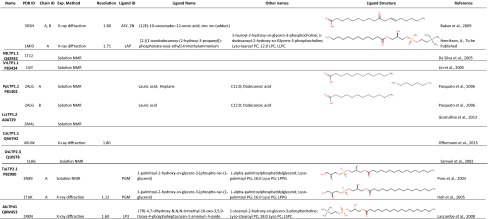
List of LTP 3D structures

TaLTP1.1 has also been crystallized binding to the fatty acid derivative prostaglandin B2 (PGB_2_) (Table 3) (Tassin-Moindrot et al. 2000). Prostaglandins are a subclass of the biologically active lipid mediators known as eicosanoids. These lipids have diverse hormone-like effects in animals. Prostaglandins are enzymatically formed from arachidonic acid, a 20-carbon unsaturated fatty acid with four *cis*-double bonds. In the prostaglandins, the carbon skeleton always contains a 5-carbon ring. The Leu77–Ile85 segment of the C-terminal part, in which the unliganded form makes contact with the H4 helix, moves outward in the solution structure of the TaLTP1.1:PGB_2_ complex [Fig. 2a, c; Tassin-Moindrot et al. (2000); (PDB ID 1CZ2)]. After binding PGB_2_, the volume of the cavity in wheat TaLTP1.1 increases from 300 ± 50 Å^3^ in the unliganded protein to 786 ± 43 Å^3^. The size of the cavity is thus comparable to the TaLTP1.1:DMPG complex. However, Tyr79 has an important role in the binding of PGB_2_. The interaction induces a 100° rotation around the Cβ–Cγ bond of the Tyr79 ring. This rotation facilitates that a hydrogen bond is formed between the carboxyl group of the ligand and the hydroxyl group of Tyr79 (Fig. 2a, c). In addition, several hydrophobic residues lining the internal cavity are pushed away by the ligand (Tassin-Moindrot et al. 2000). The most drastic conformational change is probably seen for Ile81, in order to avoid unfavorable contacts with the hydroxyl group of the aliphatic chain of PGB_2_ (Fig. 2a, c). The comparisons of these TaLTP1.1 structures show clearly that Leu77, Tyr79, and Ile81 in the C-terminal part adopt their conformation and position depending on the size and chemical nature of the ligand (Fig. 2d).

Another approach for studying lipid:protein interactions is to monitor the change in intrinsic fluorescence of tyrosine residues in the protein after addition of a lipid ligand (Douliez et al. 2000). According to such experiments on wheat TaLTP1.1, the dissociation constant and stoichiometry were fairly constant from C14 to C18 chain lengths with a *K*_d_ between 0.3 to 0.7 μM and approximately 1.7 bound ligands per protein (Douliez et al. 2000). Furthermore, the affinities of wheat LTP1 for *cis*- or *trans*-unsaturated C18 fatty acids were quite similar to the affinity for the saturated stearic acid.

### Maize LTP1

**Fig. 3:**
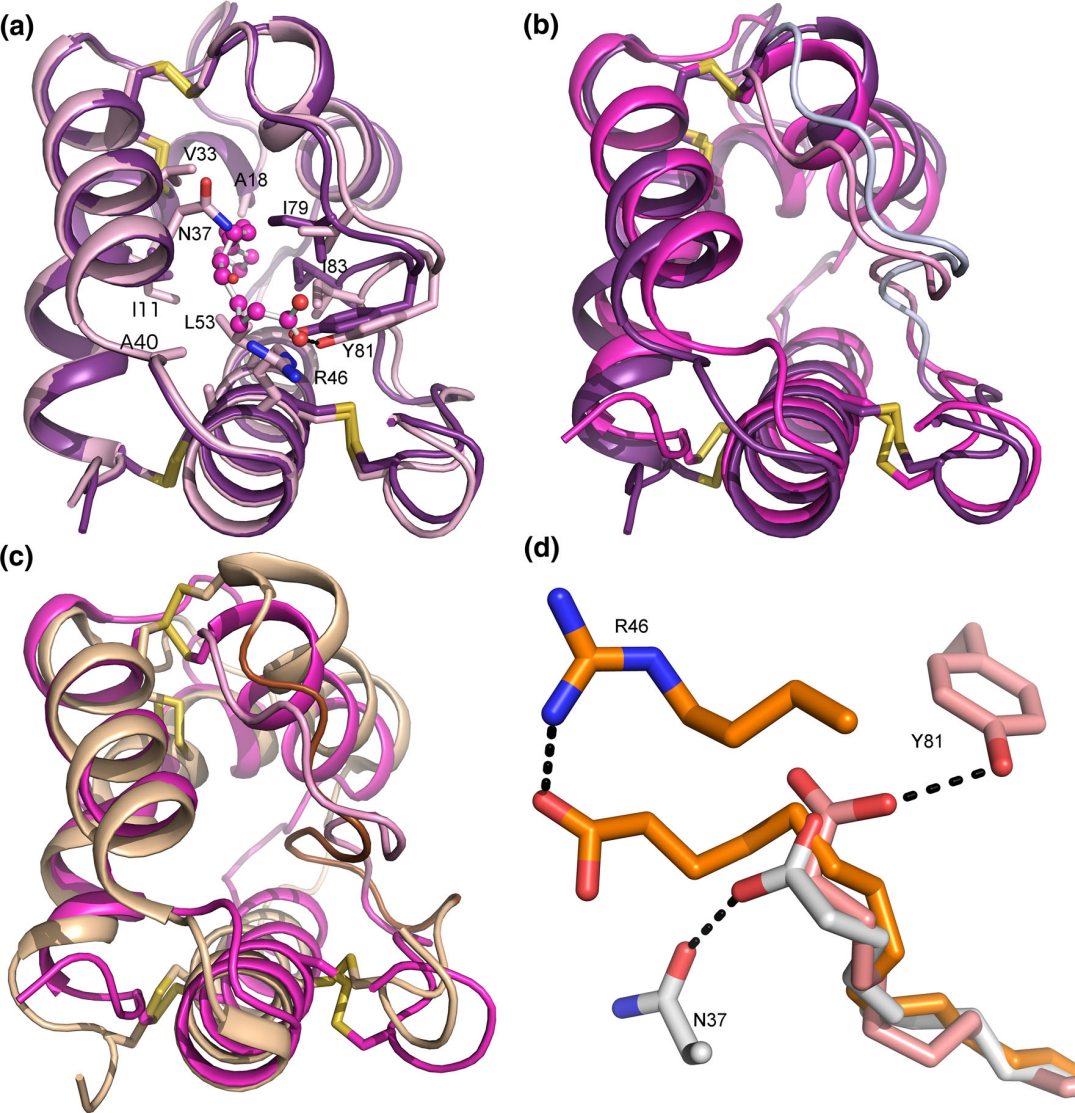
The 3D structure of ZmLTP1.6. **a** ZmLTP1.6 without ligand (*dark violet*) and with palmitic acid (*light pink*; palmitic acid shown as *pink ball-and-stick*). Residues that change their position most are shown as sticks. **b** The NMR (*magenta*) and X-ray (*dark violet*) unliganded structures of ZmLTP1.6. Differences between the structures are mainly located to the C-terminal region (residues 75–84 shown with *lighter colors*). **c** The NMR structures of ZmLTP1.6 (*magenta*) and TaLTP1.1 (*wheat* and *brown*). Obvious differences between the structures are found in the positions of helices H1 and H4, the loops, and the C-terminal region. **d** The fatty acid binding properties of ZmLTP1.6. The carboxyl groups of oleic acid (*orange*), myristic acid (*white*), and palmitoleic acid (*pink*) form a hydrogen bond with Arg46 (*orange*), Asn37 (*white*), and Tyr81 (*pink*), respectively

The first high-resolution crystal structure of any plant LTP1 was published 1995, when the 3D structures of unliganded and palmitate-bound maize ZmLTP1.6 were determined at 1.9 Å (PDB ID 1MZL) and 1.8 Å (PDB ID 1MZM) resolution, respectively (Shin et al. 1995) (Table 3). Similar to wheat TaLTP1.1, in maize ZmLTP1.6 both the N-terminal and C-terminal regions are linked to the long helix H3, by a pair of disulfide bonds, namely, Cys4–Cys52 and Cys50–Cys89. The other pairs Cys14–Cys29 and Cys30–Cys75 link the ends of helices H1B and H4 to the N-terminus of another long helix, H2 (Fig. 3a; dark violet). The volume of the hydrophobic cavity, which runs through the protein, was estimated to 300 Å^3^. One end of the tunnel, near Ala40, has an opening of 5 Å in diameter, while the other end of the tunnel, near Ala18, has a narrower opening with a diameter of 3 Å. There are polar and charged residues in the vicinity of the larger opening, while only non-polar residues nearby the smaller opening (Fig. 3a).

In the ZmLTP1.6:palmitic acid complex, there are structural changes in the C-terminal region that result in a slight swelling of the cavity (Fig. 3a). The residues Ile11, Ile79, Tyr81, and Ile83 are displaced from the hydrophobic cavity to let the palmitate acyl-chain fit inside the cavity, while its carboxyl group forms a hydrogen bond with the hydroxyl group of Tyr81. Apart from these changes, the overall fold of the complex is identical to the uncomplexed structure. Furthermore, only one acyl chain could fit into the cavity of the maize ZmLTP1.6 according to Shin et al. (1995). Binding of another chain would mean that the second one has to extend into the solvent.

The 3D solution structure of ZmLTP1.6 (Gomar et al. 1996; PDB ID 1AFH) was published shortly after the publication of the 3D crystal structure. The solution and crystal structures showed good correlation with clear differences only in the C-terminal region (Fig. 3b). Comparison of the solution structures of TaLTP1.1 and ZmLTP1.6 revealed differences in helices H1 and H4 (Fig. 3c). H1 is somewhat longer in ZmLTP1.6, while H4 in TaLTP1.1 is disrupted by two consecutive prolines.

Incubation of maize ZmLTP1.6 with 16(9-anthroyloxy)palmitate either alone or together with another fluorescent fatty acid analog, P-96, revealed that the binding cavity of ZmLTP1.6 can accommodate two fatty acids simultaneously (Zachowski et al. 1998). Further competition experiments with anthroyloxy-fatty acid analogs showed that fatty acids of 16–19 carbons were the preferred ligands. Fatty acyl-CoA or LPC derivatives bound as well as the corresponding fatty acids. The presence of one double bond did not change appreciably the affinity of ZmLTP1.6, while the presence of two or three double bonds or of a hydroxyl moiety significantly reduced the affinity.

Lipid transfer assays, where the transfer of labeled lipids from quenched donor vesicles to unquenched acceptor vesicles is measured, are also frequently used to investigate the properties of LTPs (Edqvist et al. 2004; Lin et al. 2005). When LTPs from wheat and maize seeds were compared in in vitro transfer assays, maize LTP had higher transfer activity and showed faster kinetics for fatty acid binding (Guerbette et al. 1999a, b).

ZmLTP1.6 was later crystallized with fatty acids of different chain lengths, from capric acid (C10:0) to stearic acid (C18:0) to investigate how the chain length would influence the interactions between protein and lipid (Han et al. 2001) (Table 3). The cavity volume of ZmLTP1.6 increases only slightly, from 558 to 582 Å^3^, when the length of the complexed fatty acid increases from C10 to C18. Furthermore, *cis*-unsaturated C18 fatty acid chains with double bonds in *cis* configuration, such as oleic acid (C18:1), linoleic acid (C18:2), and linolenic acid (C18:3), were also used as ligands during crystallization. Double bonds in *cis* configuration enforce a more curved shape on the fatty acid compared with the saturated fatty acids which are linearly shaped. On the other hand, fatty acids with double bonds in *trans*-configuration are linear and more similar to the saturated fatty acids. Therefore, the LTPs could possibly show different binding modes or affinities for saturated, *cis*- or *trans*-fatty acids. The maize ZmLTP1.6 was also crystallized with the hydroxylated, *cis*-unsaturated C18 fatty acid 12-hydroxy-9-*cis*-octadecenoic acid (ricinoleic acid). The double bond and the hydroxyl group give the ricinoleic acid a more bulky shape compared to the other more common C18 fatty acids. Ricinoleic acid is the major component of the seed oil obtained from *Ricinus communis* L. (castor oil plant).

The crystals of the ZmLTP1.6:ligand complexes revealed that the cavity volume somewhat depends on the shape of the C18 fatty acid, expanding from 557 Å^3^ for stearic acid up to 620 Å^3^ for ricinoleic acid (Han et al. 2001). This implies that there is a requirement for lipid-dependent plasticity in the shape of the cavity. In several of the ZmLTP1.6:ligand complexes, the ligands bind favorably into the cavity in only one of two possible directions due to the interactions with Tyr81, Arg46, and Asn37 (Fig. 3d). These residues are located along the top opening of the cavity and interact with the carboxylate group of most ligands. For instance, the carboxyl group of the shorter fatty acids, lauric acid (C12:0; PDB ID 1FK1), and myristic acid (C14:0¸ PDB ID 1FK2) (Table 3) forms a hydrogen bond with the side chain of Asn37, whereas the carboxyl group of the longer C16 fatty acids, palmitic acid (C16:0; PDB ID 1MZM), and palmitoleic acid (C16:1; PDB ID 1FK3) forms a hydrogen bond with the hydroxyl group of Tyr81 (Fig. 3d). However, the ZmLTP1.6 complexes with capric acid (C10; PDB ID 1FK0) and oleic acid (C18:1; PDB ID 1FK3) have two different conformations where the carboxylate group of the fatty acids is located either close to the top or to the bottom opening of the cavity (Han et al. 2001). In conformation 1 of the ZmLTP1.6:oleic acid complex, the O1 atom of the oleate carboxylate group forms a hydrogen bond with the NH2 group of Arg46 (Fig. 3d), and the O2 atom of the carboxylate group donates the proton to the main chain oxygen atom of either Asn40 or Ala37. In the ZmLTP1.6:oleic acid complex conformation 2, the O1 and O2 atoms of the carboxyl group in oleate interact with the hydroxyl group of Tyr81. On the other hand, the carboxylate group of capric acid does not form any hydrogen bonds with ZmLTP1.6.

Several LTP1s, such as those from Arabidopsis, cabbage, and maize, have been shown to bind with calmodulin, which is a ubiquitous Ca^2+^-binding protein (Li et al. 2008; Shang et al. 1991; Wang et al. 2005). When interacting with maize ZmLTP1.2, calmodulin seems to inhibit the lipid binding activity of LTP according to the result from an assay based on binding to P-96 (Li et al. 2008). Curiously, calmodulin has the opposite effect on the *Brassica rapa* subsp. *Pekinensis* (chinese cabbage) BrLTP1.9 (BP-10), as binding to calmodulin enhances its P-96 binding activity.

### Rice LTP1

The crystal structure of unliganded rice OsLTP1.18 at 1.6 Å resolution (PDB ID 1RZL) showed a fold very similar to maize ZmLTP1.6 (Lee et al. 1998). Anyway, two regions with clear differences can be identified. First, the deletion of Gln21 in ZmLTP1.6 results in a large displacement of the residues 19–22 in the loop between H1 and H2. Second, in OsLTP1.18, the C-terminal loop around Tyr79 is collapsed into the hydrophobic cavity, which leads to a considerably smaller cavity, calculated to 144 Å^3^ for OsLTP1.18 (Lee et al. 1998). In both the X-ray and the NMR structures of OsLTP1.18 (PDB ID 2BV2; Poznanski et al. 1999), the side-chain of Arg44 swings down toward the cavity and partially plugs the opening. The side-chain of Ile81 terminates the other end of the cavity, while the side-chain of Tyr79 divides the cavity into two parts.

**Fig. 4:**
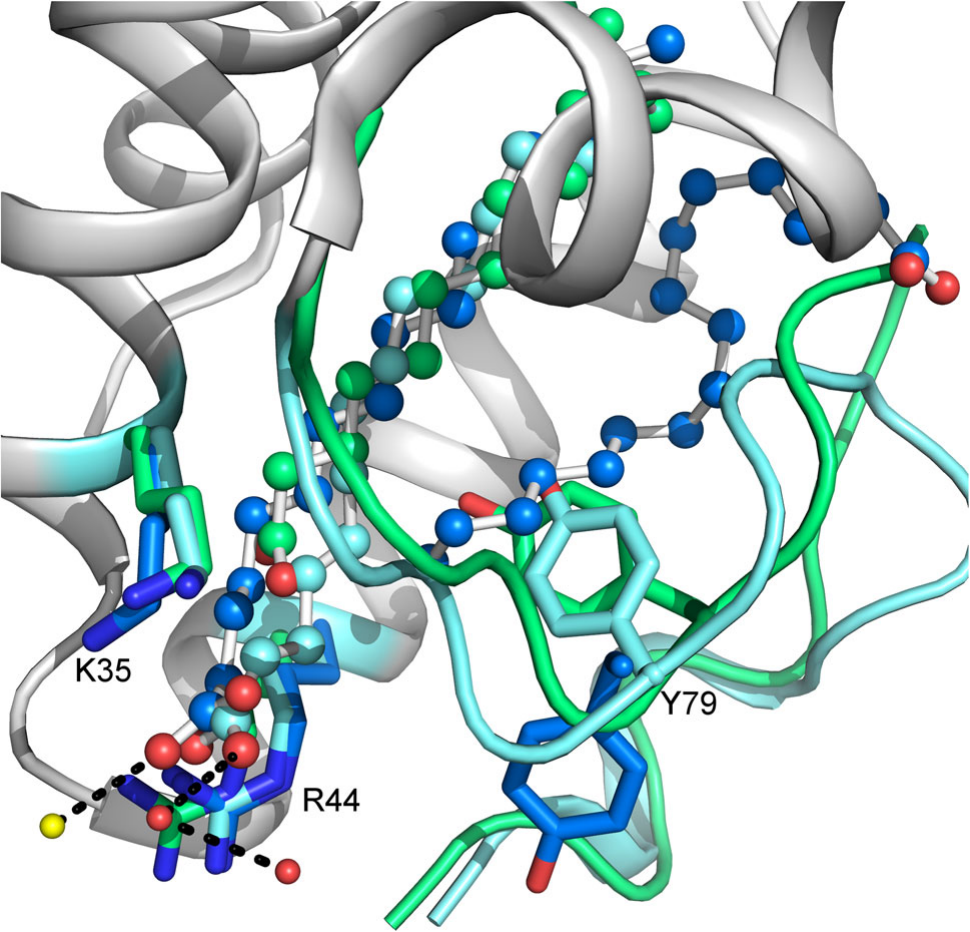
OsLTP1.18 in complex with myristate (*cyan*), two palmitates (*blue*), and stearate (*green*). Tyr79 swings away from the lipid binding cavity when the protein accommodates the second palmitate molecule. Lys35 and Arg44 create a positively charge environment in the cavity opening, but they are not involved in direct hydrogen bonds with the lipids. Similarly, the carboxyl group of stearate is nearby Tyr79, but the bonding distance is too long for a hydrogen bond. The C-terminal region (*green*) adopts slightly different conformation in the stearate complex compared with the two other complexes (*cyan*). Both myristate and palmitate interact with water molecules that surround the protein. The water molecules involved in myristate binding are shown as *red spheres* and the one interacting with palmitate as a *yellow sphere*

The X-ray structures of OsLTP1.18 in complex with myristic acid (PDB ID 1UVA), palmitic acid ((PDB ID 1UVB), and stearic acid (PDB ID 1UVC) (Table 3) revealed that the ligand binding required a noteworthy swelling of the cavity. During the ligand binding, Arg44 moves away from the cavity, and it is, therefore, not involved in forming hydrogen bonds. Rather, Arg44 acts as a gate keeper giving the lipids access to the tunnel. Similarly, Tyr79 moves away from the lipid to create a binding site in the cavity. The distances between the hydroxyl group of Tyr79 and the carboxyl group of lipid, thereby, become too large to enable the formation of hydrogen bonds (Fig. 4). Rather than interacting with the protein, both myristic acid and palmitic acid interact with water molecules surrounding the protein (Cheng et al. 2004b).

### Barley LTP1

**Fig. 5:**
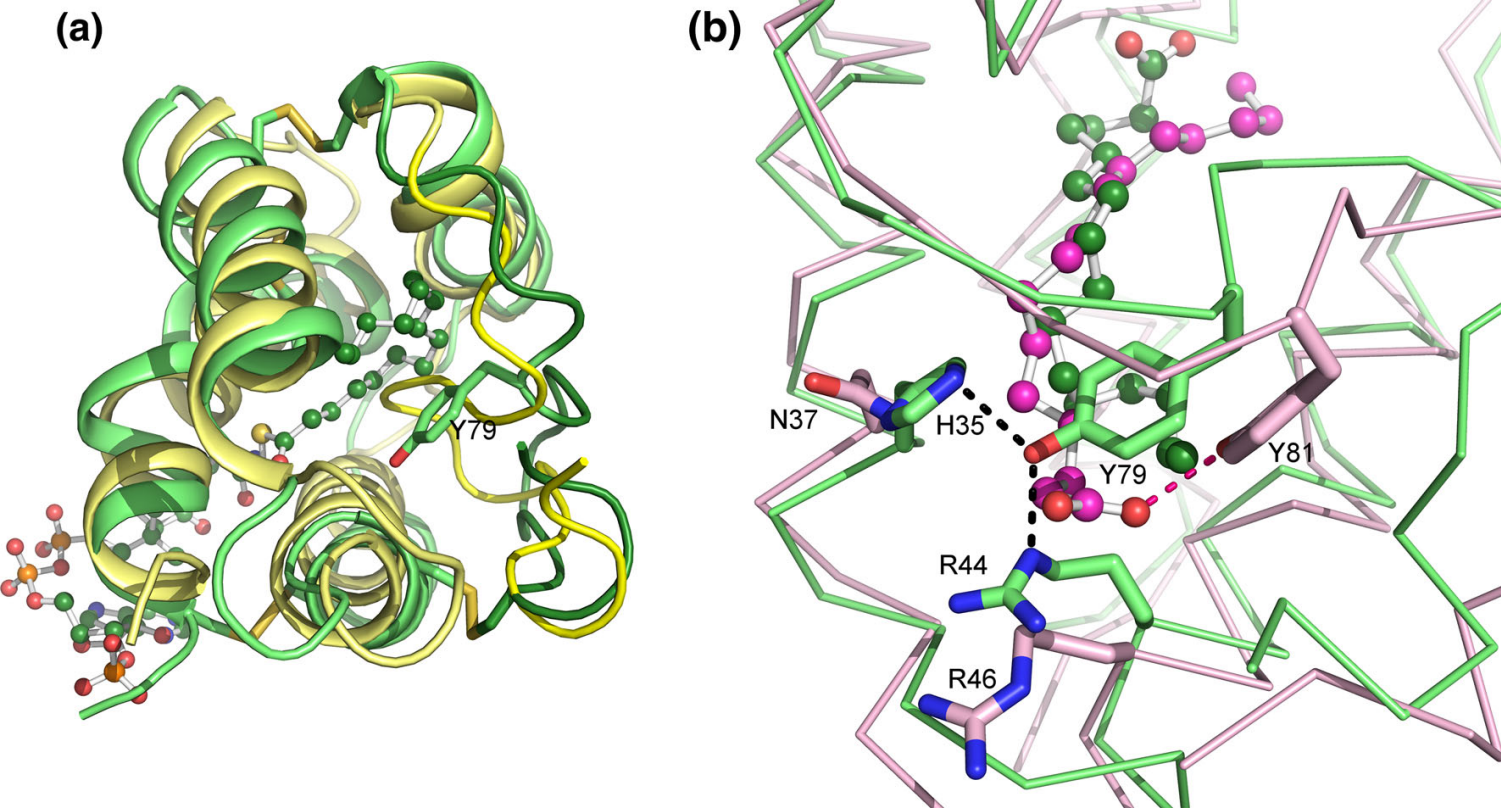
The 3D structure of barley HvLTP1.1. **a** The large structural differences that occur in HvLTP1 upon binding of PCoA (*green ball-and-sticks*). The HvLTP1.1:PCoA complex is superimposed on the unliganded form (*yellow*) of HvLTP1.1. Major conformational changes occur in the C-terminal part of HvLTP1.1. **b** The HvLTP1.1:palmitate complex (*green*) superimposed on the ZmLTP1.6:palmitate complex (*violet*). In the HvLTP1.1:palmitate complex, carboxyl group of the palmitate (shown as *green ball-and-sticks*) does not interact with Tyr79. Instead, Tyr79, Arg44 and His35 form hydrogen bonds with each and close the cavity opening. The orientation of palmitate is opposite to that in the ZmLTP1.6 complex (*violet*, palmitate shown in *magenta*) where palmitate interacts with Tyr81 (*red bond*)

Acyl-coenzyme As (acyl-CoAs) are coenzymes where fatty acid is linked to the terminal thiol moiety of CoA. The acyl-CoAs are commonly involved in metabolism of fatty acids and other lipids, such as in β-oxidation and glycerolipid synthesis. Several acyl-CoAs have been used in the crystallization and specificity studies of the LTPs. For instance, barley HvLTP1.1 was crystallized in a complex with palmitoyl-CoA (PCoA) (Lerche et al. 1997). The solution structures of the uncomplexed barley HvLTP1.1 (PDB ID 1LIP; Heinemann et al. (1996) and the HvLTP1.1:PCoA complex (PDB ID 1JTP; GI:157830246) unveiled a major conformational change in the protein upon ligand-binding (Lerche et al. 1997). The cavity volumes were calculated to 39 Å^3^ for the uncomplexed HvLTP1.1 and 620 Å^3^ for the LTP:PCoA complex (Lee et al. 1998). This expansion of the cavity is obtained by a bend in helix H1 and by conformational changes in both the C-terminus and helix H3 (Fig. 5a). The palmitoyl chain of PCoA is completely buried in the hydrophobic cavity, where it is bent in a U-shape. Met10, in H1, and Tyr79, in the C-terminal part, are two key residues that interact with each end of the palmitoyl chain.

The binding of palmitic acid causes much less of structural alterations in HvLTP1.1. In this case, the protein undergoes significant structural perturbations only in the C-terminal residues (Lerche and Poulsen 1998). The modes for binding palmitic acid are different between maize ZmLTP1.6 and HvLTP1.1. In the ZmLTP1.6 complex, the carboxyl end of palmitic acid is in close vicinity to Arg44 and Tyr79, and the methyl group makes contacts to the hydrophobic residues in the second half of H1 and H4. In the HvLTP1.1:palmitate complex, the fatty acid is oriented in the completely opposite direction (Fig. 5b). Molecular simulations suggest that a range of small sequence differences in the H1–H2 loop, connecting H1 and H2 at the base of the hydrophobic cavity, and in H1 contribute to the different binding modes in barley HvLTP1.1 and maize ZmLTP1.6 (Smith et al. 2013).

The intrinsic fluorescence of tyrosine was used to probe the binding of lipids to HvLTP1.1. However, at first, the solvent exposed Tyr91 had to be removed from HvLTP1.1 by cleavage with carboxypeptidase. The K_d_ for binding to LMPC for this truncated form of HvLTP1.1 was close to 10^−6^ M (Douliez et al. 2001), which is similar to the *K*_d_ reported for wheat TaLTP1.1 (Douliez et al. 2000). HvLTP1.1 was also shown to bind to ω-hydroxypalmitate with a *K*_d_ comparable to what was found for LMPC. Titrations with LMPC further revealed that barley HvLTP1.1 could bind two LMPC molecules simultaneously (Douliez et al. 2001).

An abundant form of LTP1, named LTP1b, with a covalently bound adduct in the form of an α-ketol has been identified in barley and wheat seeds (Perrocheau et al. 2006; Douliez et al. 2001). The α-ketol adduction enhances the lipid transfer activity of both the wheat and barley LTP1s, as revealed in a transfer assay using donor vesicles containing pyrene-PG. In the crystal structure of barley HvLTP1b (PDB ID 3GSH), the α-ketol is partly exposed at the surface of the protein and partly buried in the hydrophobic cavity (Bakan et al. 2006).

### Other LTP1

Tobacco NtLTP1.1 was produced in *Pichia pastoris* from a cDNA isolated from the shoot apex of tobacco, and its 3D structure was investigated with NMR spectroscopy (Da Silva et al. 2005). The global fold of the NtLTP1.1 (PDB ID 1T12) is very similar to that of cereal seed LTP1. The cavity volume of NtLTP1.1 was calculated to 318 Å^3^. The binding properties of NtLTP1.1 were analyzed by following the chemical shift variations of NMR signals upon lipid binding. These measurements indicated that only one LMPC molecule could fit into the hydrophobic cavity. Possibly, this is due to a cluster of hydrophobic residues close to the second possible entrance to the cavity. Addition of LMPC induces a noticeable shift of Tyr79 resonances, indicating that binding is associated with a structural change around Tyr79. NtLTP1.1 was also found to bind to palmitate and oleate, as measured by tyrosine fluorescence. The *K*_d_ was determined to 0.5 μM for LMPC, 5.6 μM for palmitate, and 3.9 μM for oleate (Da Silva et al. 2005).

Lipid-binding assays based on the displacement of the fluorescent TNS from the hydrophobic cavity of NtLTP1.1 showed that the ligands could be placed in three groups based on the TNS displacement efficiency. The *cis*-unsaturated linoleic acid and oleic acid gave highly efficient displacement, and medium efficient displacement was shown for two other *cis*-unsaturated fatty acids:palmitoleic acid and linolenic acid, as well as for the oxylipin jasmonic acid. Low or no displacement was shown for saturated fatty acids and the *trans*-unsaturated elaidic acid (Buhot et al. 2004).

Similar results were obtained with GbLTP1.1 from the non-flowering seed plant *Ginkgo biloba* when its lipid binding capacity was assayed with the TNS displacement approach. The GbLTP1.1 was originally purified from seeds, but for the studies on lipid-binding, the GbLTP1.1 was expressed in *E. coli* as a thioredoxin-fusion. The reduction in fluorescence showed that *cis*-unsaturated fatty acids, such as palmitoleic acid, oleic acid, linoleic acid, and linolenic acid, could displace TNS from the binding cavity in the Ginkgo LTP1. In contrast, saturated fatty acids (C8:0–C18:0) and the *trans*-unsaturated elaidic acid could not compete with TNS (Sawano et al. 2008).

The *Vigna radiata* (mung bean) VrLTP1.1 was purified from seeds, and its 3D structure was determined by solution NMR spectroscopy (PDB ID 1SIY). Comparison of VrLTP1.1 and rice OsLTP1.18 showed that conformational changes of the C-terminal loop of VrLTP1.1 result in a larger hydrophobic cavity volume. The volume of the hydrophobic cavity in VrLTP1.1 is 510 ± 45 Å^3^, while it is only 330 ± 44 Å^3^ for rice OsLTP1.18. Nevertheless, VrLTP1.1 and OsLTP1.18 showed very similar activities when tested in a lipid transfer assay based on monitoring the increase in fluorescence resulting from the transfer of pyrene-PC from quenched donor vesicles to unquenched acceptor vesicles (Lin et al. 2005).

*Prunus persica* (peach) PpLTP1.1 (Pru p 3) was crystallized in complex with a ligand, presumably a fatty acid resembling laurate originating from the heterologous production in *E. coli* (Pasquato et al. 2006; (PDB ID 2ALG; PDB ID 2B5S). Two molecules of PpLTP1.1 were found that bound the ligand in different ways. One molecule (Molecule A) is the fully liganded protein, while the other molecule (Molecule B) represents a partially ligated state. The most significant difference between the molecules is found in two regions formed by residues 52–58 and 76–85, respectively. The former corresponds to the final part of the α-helix 3 and the loop connecting it to helix 4, and the latter is close to the C-terminus. In Molecule B, the latter region collapses toward the core of the molecules leading to a reduction in the size of the cavity. Tyr79 is playing a significant role, as its side-chain is on the external surface in the case of Molecule A and points toward the interior cavity in Molecule B, occupying part of the space of ligand bound in molecule A. In barley HvLTP1.1 complexed with a ligand, Tyr79 is oriented as in Molecule A, while in liganded wheat TaLTP1.1, rice OsLTP1.18, and maize ZmLTP1.6, the Tyr79 points toward the interior of the cavity as in Molecule B.

Superpositioning of the liganded PpLTP1.1 with the crystal structure of the unliganded *Corylus avellana* (hazelnut) CaLTP1.1 (Cor a 8) revealed striking differences in the binding pocket. In the liganded PpLTP1.1, lauric acid occupies the binding cavity, whereas in the unliganded CaLTP1.1, the cavity is occupied by Tyr103 (corresponding to Tyr79 of PpLTP1.1) (PDB ID 4XUW; Offermann et al. 2015).

The *Lens culinaris* (lentil) LcLTP1.2 (Lc-LTP2) was produced as a thioredoxin fusion in *E. coli*, and its 3D structure in solution was obtained with NMR (PDB ID 2MAL; Gizatullina et al. 2013). LcLTP1.2 resembles other LTP1s with four helices surrounding a hydrophobic cavity. In the unliganded state, the LcLTP1.2 holds a rather large cavity with a volume of approximately 600 Å^3^. NMR spectroscopy revealed that upon binding to DMPG the cavity expands to enable the accommodation of the double chained lipid. Interestingly, the DMPG:Lc-LTP2 complex have only rather limited lifetime with a half-life of about 40 h.

### Rice LTP2

**Fig. 6:**
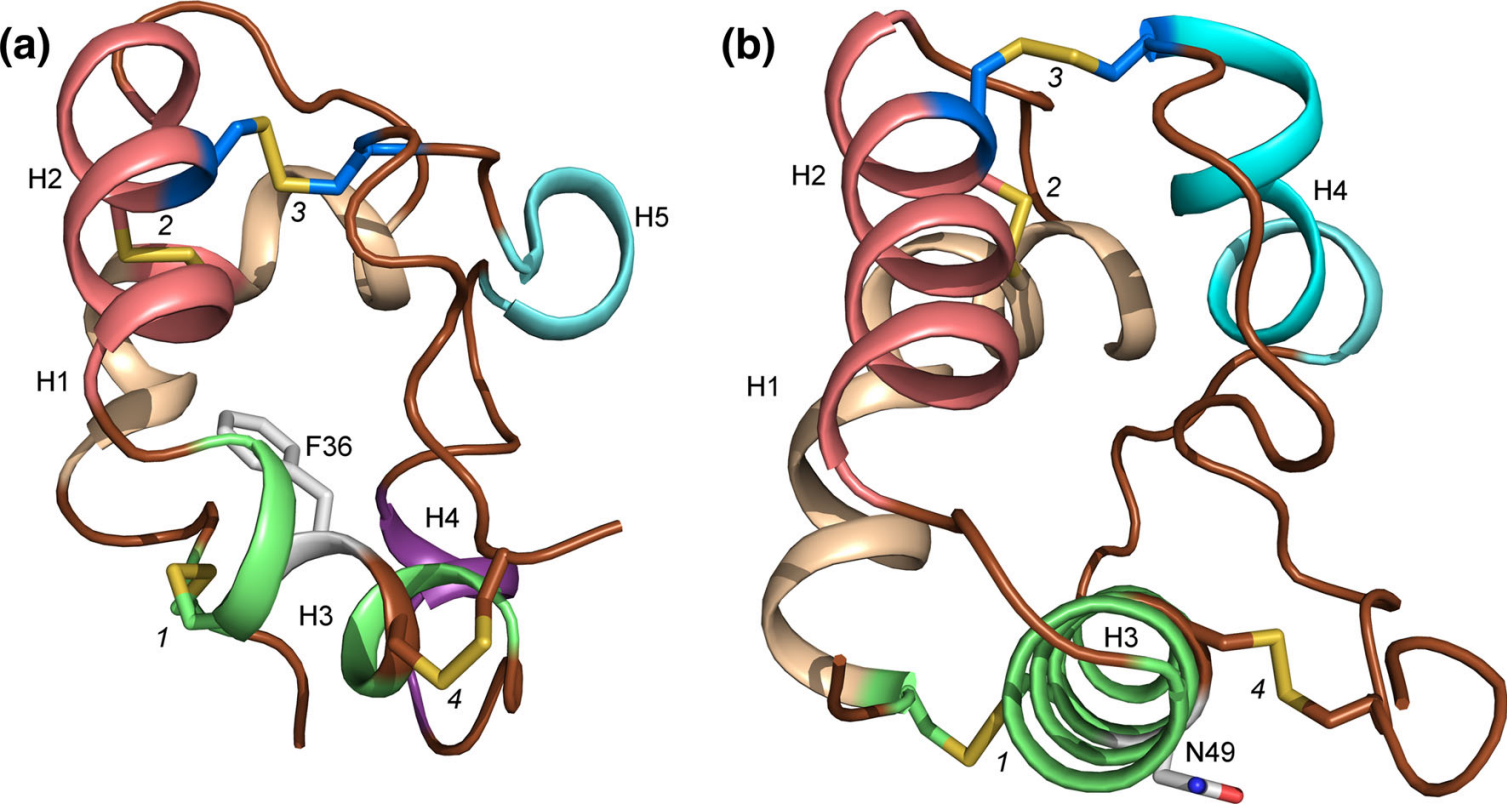
Comparison of the 3D structures of rice LTP2 (OsLTP2.3) and LTP1 (OsLTP1.18). **a** The NMR structure of rice OsLTP2.3. The first and fourth disulfide bridges differ from LTP1 and are formed between C^1^–C^5^ and C^6^–C^8^. Due to this difference, Phe36 (*white sticks*) in the C^5^XC^6^ motif points to the ligand binding cavity. **b** The NMR structure of rice OsLTP1.18. The four disulfide bridges formed by C^1^–C^6^, C^2^–C^3^, C^4^–C^7^, and C^5^–C^8^ are labeled. Asn49 (*white sticks*) in the C^5^XC^6^ motif is located on the surface

The solution structure of OsLTP2.3 purified from rice flour was published in 2002 (PDB ID 1LH6; Samuel et al. 2002). The 3D-fold of OsLTP2.3 consists of five α-helices and, similar to LTP1, eight cysteines form four disulfide bonds to stabilize the structure. In OsLTP2.3, the pairing occurs between the cysteines Cys13–Cys35, Cys11–Cys25, Cys26–Cys61, and Cys37–Cys68. Thus, the disulfide bridges are formed between C^1^–C^5^, C^2^–C^3^, C^4^–C^7^, and C^6^–C^8^ of the 8CM in LTP2, whereas in LPT1, C^6^ is paired with C^1^ and C^5^ with C^8^. Therefore, the first and fourth bridges differ between LTP1 and LTP2. Furthermore, between the 3D structures of rice OsLTP2.3 and OsLTP1.18, there is a major difference in the position of residue X in C^5^XC^6^ of the 8CM. In rice OsLTP2.3, this residue is a hydrophobic Phe buried inside the protein (Fig. 6a), whereas in rice OsLTP1.18, the corresponding polar Asn is projected toward the surface of the protein (PDB ID 2BV2; Poznanski et al. 1999) (Fig. 6b). This difference may in part explain the different shapes of the hydrophobic cavities in OsLTP2.3 and OsLTP1.18. Samuel et al. (2002) described the shape of the OsLTP2.3 cavity as a triangular hollow box, while the shape is more tunnel-like in LTP1. The volume of the cavity in OsLTP2.3 was measured to be 140 Å^3^ and, thus, somewhat smaller than in most LTP1s. Molecular modeling suggested a high degree of flexibility concerning the size and shape of the cavity, such that the binding of one molecule of stearate would increase the cavity volume to 825 Å^3^.

OsLTP2.3 efficiently transfers lipid molecules between vesicles despite its smaller cavity (Samuel et al. 2002). Interestingly, rice OsLTP2.3, but not rice OsLTP1.18, binds to dehydroergosterol (DHE), a cholesterol analog with intrinsic fluorescence (Cheng et al. 2004a). The *K*_d_ for binding to DHE by rice LTP2 was measured to 71 µM. Tyr45 at the opening of the cavity seems to be critical for the lipid binding and transfer in OsLTP2.3. A Tyr45Ala mutant has similar 3D structure as the wild-type (WT) protein. However, it has a severely reduced capacity for binding to LMPC and DHE and also a lowered activity compared to the WT protein in lipid transfer assays (Cheng et al. 2008). Docking analysis indicated that Tyr45 directly interacts with LMPC as well as being involved in hydrophobic interactions with several carbon atoms in residues 39, 42, 44, 46, and 49. Other residues in OsLTP2.3 important for lipid binding are Ile15 and Tyr48, which both are located at the opening of the cavity. Ile15 may be involved in controlling the entry of the sterol to the cavity, while Tyr48 is important for planar sterol binding (Cheng et al. 2008).

### Wheat LTP2

**Fig. 7:**
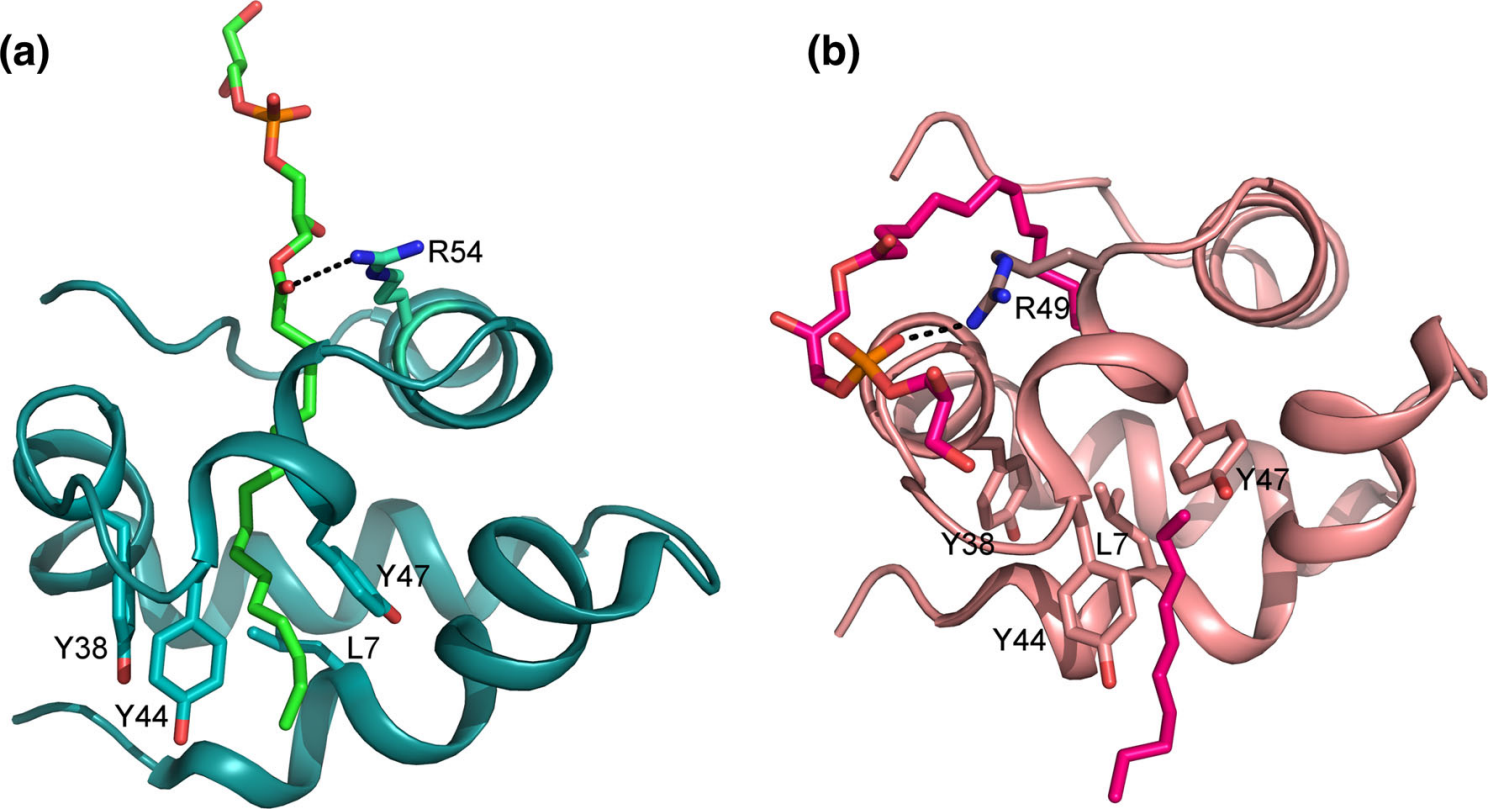
3D structure of the wheat LTP2 TaLTP2.1. **a** The NMR structure of TaLTP2.1 (*cyan*) in complex with LPPG (shown as *green sticks*). Arg54 makes a hydrogen bond with LPPG, which is bound in a continuous cavity. Residues Leu7, Tyr38, Tyr44, and Tyr47 in the distal opening are shown as *sticks*. **b** The X-ray structure of TaLTP2.1 (*pink*) in complex with two LPPG molecules (*magenta*). One of the LPPG molecules forms a hydrogen bond with Arg49 instead of Arg54

The solution structure of wheat TaLTP2.1 in complex with LPPG (PDB ID 1N89; Pons et al. 2003) (Table 3) revealed a structure consisting of five helices arranged in a superhelical tertiary structure. The cavity volume (341 Å^3^) of TaLTP2.1 is in the same range as TaLTP1.1, although TaLTP2.1 is shorter by 24 residues (Pons et al. 2003). Only one unique phospholipid position was found for LPPG in all retained solution structures of TaLTP2.1 (Fig. 7a). The fatty acid chain is completely embedded in the protein, and the terminal methyl group of the fatty acid chain is positioned between the H1 and H4 helices. The proximal entrance of the cavity, where the phosphate group of the lipid is found, is characterized by several hydrophilic and basic residues; Arg49, Arg54, Thr58, and His66. The distal opening of the cavity presents hydrophobic residues, such as Leu7, Tyr38, Tyr44, and Tyr47.

The crystal structure of the TaLTP2.1 in complex with LPPG showed two independent ligand binding sites (Fig. 7b; PDB ID 1TUK; Hoh et al. 2005). The major lipid-binding site is the large and long cavity, with the shape of an elongated curved channel of about 17 Å length and 5 Å in diameter with a volume of 300 Å^3^, and the minor cavity has a volume of 130 Å^3^. In the X-ray structure, the residues Leu7, Ile14, and Leu28 form the bottom of the main cavity and define the wall to the minor cavity (Hoh et al. 2005), whereas in the solution structure, they have a different orientation that allows the formation of one continuous cavity (Pons et al. 2003).

### Arabidopsis DIR1

**Fig. 8:**
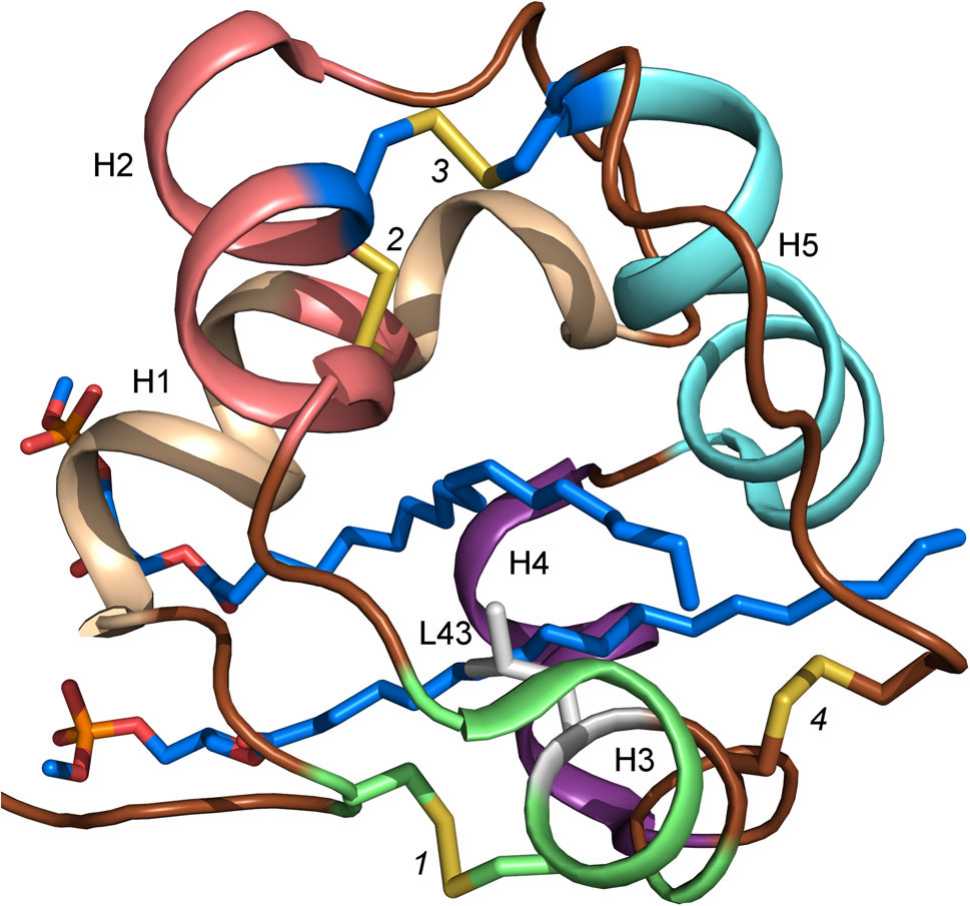
The X-ray structure of DIR1. The disulfide bonds are formed similarly as in LTP2s. The residue in the C^5^XC^6^ motif is the buried and hydrophobic Leu43. The lipid binding site accommodates two LSPC (*blue sticks*) in the binding site

The Arabidopsis AtLTPd1 (DEFECTIVE IN INDUCED RESISTANCE; DIR1) was crystallized in complex with two molecules of LSPC (Table 3). DIR1 follows the general LTP-fold, with five helices connected by four disulfide bonds arranged in a super-helical pattern around a central tunnel-shaped cavity (Fig. 8; PDB ID 2RKN; Lascombe et al. 2008). After an elongated N-terminal segment followed by a turn, the DIR1 structure begins with a long α-helix (H1). Three residues in 3/10-helix conformation complete this first α-helix. In wheat TaLTP2.1, the 3/10 helix forms an angle of ~90° with the H1, while in DIR1, H1 and the second 3/10 helix are almost collinear. This opens up the central channel of DIR1, allowing entry and room for two lipid molecules. The volume of the cavity is 242 Å after removing the two lipids. The cavity is fully lined with hydrophobic residues, while some polar residues are located around the large tunnel entrance. The C-terminal segment has no defined secondary structure, except for the last residue, Cys77, which is involved in a disulfide bond.

In DIR1, the four cysteine pairs are Cys5–Cys42, Cys15–Cys31, Cys32–Cys69, and Cys44–Cys77 accordingly to what is found for the LTP2-family. Moreover, the size of DIR1 is also closer to LTP2 than to LTP1. Similar to the solution structure of wheat TaLTP2.1 (Fig. 7a; Pons et al. 2003), in DIR1, the two bound LSPC molecules are fully extended, arranged side by side, parallel to each other in the same cavity (Fig. 8). On the other hand, in the X-ray structure of wheat TaLTP2.1, the two ligands are located in two different compartments (Fig. 7b; Hoh et al. 2005).

When the lipid binding of DIR1 was tested with LPC derivatives carrying acyl chains of different lengths, it was more efficiently binding longer fatty acyl chains (C18) than shorter chains (C14) (Lascombe et al. 2008). The *K*_d_ for binding to LMPC was 0.3 μM, for LPPC 0.03 μM, and for LSPC 0.06 μM. These data may be compared to wheat TaLTP1.1 for which the *K*_d_ values for binding to the LMPC, LPPC, and LSPC are 0.4, 0.7, and 0.7 μM, respectively (Douliez et al. 2000).

### Physcomitrella LTP

The structure has not been determined experimentally for any LTPs from early diverging land plants, such as mosses or livermosses. Molecular modeling suggests that LTPds and LTPgs from the livermoss *Marchantia polymorpha* and the moss *P. patens* have similar 8CM and disulfide bond patterns as LTPd and LTP2 (Edstam et al. 2011). When the lipid binding of two LTPgs from *P. patens* was tested in a TNS competition assay with saturated and *cis*-unsaturated C18 fatty acids, both PpLTPg2 and PpLTPg8 showed a preference for *cis*-unsaturated fatty acids (Edstam et al. 2014). The competition assay further revealed that the moss LTPGs were more readily binding to stearoyl-CoA compared to stearate. The ω-hydroxy fatty acid 22-hydroxydocosanoic acid was found to compete with less efficiency for binding to the PpLTPgs than oleic acid, linoleic acid, and stearoyl CoA. The ω-hydroxy fatty acids are major components of plant surface polyesters, such as suberin and cutin, and could, therefore, possibly be a natural ligand for the LTPs.

## Functional investigations of LTPs

There are numerous reports demonstrating the expression pattern of individual LTPs (Boutrot et al. 2007; Li et al. 2014a; Wei and Zhong 2014; Yu et al. 2014). One conclusion that can be drawn from these experiments is that LTPs are abundantly expressed in all tissues of the plant. To take the expression analysis further, microarray data were exploited and analyzed for coexpression patterns of LTPg genes in rice and Arabidopsis. The results showed that based on coexpression LTPgs can be arranged in three clusters (AtI-III and OsI-III). Each expression cluster contains 3–8 LTPg genes. In one cluster from each plant, AtI in Arabidopsis and OsI in rice, the expression is restricted to aerial parts of the plant. The second cluster, AtII or OsII, is the only one with expression in roots, while expression of the third cluster, AtIII or OsIII, is restricted to reproductive tissues. Gene ontology analyses of the Arabidopsis clusters indicate that the AtI is primarily involved with cuticular wax accumulation, AtII with suberin synthesis or deposition and AtIII with sporopollenin accumulation (Edstam et al. 2013). Thus, there are defined clusters of LTPs with common expression patterns, at least in both Arabidopsis and rice. Each LTP cluster likely operates to complete a specific biological process.

### LTPs play a role in signaling

It has been rather challenging to connect LTP knock-downs or knock-outs with phenotypes, probably due to a high degree of gene redundancy. However, since the first phenotype for an LTP mutant was reported for about 15 years ago, there has been a slow but steady accumulation of LTP-related phenotypes. The wide array of phenotypes reported reveals that the LTPs play important roles in many different tissues and organs of plants.

The Arabidopsis AtLTPd1 (DIR1) was the first LTP where a mutation could be connected to a phenotype. The analysis of the *dir1*-*1* mutant revealed a role in systemic resistance signaling for DIR1 (Maldonado et al. 2002). The *d* *ir1*-*1* plants exhibit WT local resistance towards infection with *Pseudomonas syringae*. However, the pathogenesis-related gene expression pattern is abolished in uninoculated distant leaves. Thus, the inoculated leaves in the *dir1*-*1* plants are defective in the production or transmission of a mobile signal.

A related phenotype is found for Arabidopsis *AZELAIC ACID INDUCED 1* (*azi1*) plants. *AZL1* (At4g12470) is encoding an LTP-like protein. It has an 8CM, but unlike the classical LTPs, there is a proline-rich region inserted between the targeting sequence and the 8CM. Azelaic acid and petiole exudates failed to induce systemic immunity in *azi1* plants, although these treatments protected WT plants against subsequent infection (Jung et al. 2009). In addition, pathogen-induced exudates from *azi1* were inactive when applied to WT plants.

Thus, like DIR1, AZI1 modulates production and/or translocation of a mobile signal during systemic acquired resistance (SAR). The phosphorylated sugar derivative glycerol-3-phosphate (G3P) is one of many chemical signals that contribute to SAR. In recent studies, it has been shown that DIR1 and AZI1 are essential for G3P–accumulation, while on the other hand, reduced levels of G3P result in decreased *DIR1* and *AZI1* transcription (Yu et al. 2013). It seems that G3P operates in a positive feedback loop with DIR1 and AZI1. The mechanistic details of the feedback loop remain unknown.

### LTPs are required for cuticular wax accumulation

AtLTPg1 (LTPG1) and AtLTPg2 (LTPG2) from Arabidopsis are both highly expressed in the epidermis of inflorescence stems and silique walls (Debono et al. 2009; Kim et al. 2012). Knock-down of LTPG1 expression results in reduced wax load on stem surfaces (Debono et al. 2009), while a *ltpg1* T-DNA knockout mutant shows a 10 % reduction of the C29 alkane (nonacosane) in stems and siliques (Lee et al. 2009). The C29 alkane is the major component of cuticular wax in stems and siliques. In a *ltpg2* knock-out mutant, the amount of the C29 alkane is reduced with 4 % in stems and 20 % in siliques (Kim et al. 2012), whereas a *ltpg1 ltpg2* double-mutant shows even further reductions of the C29 alkane. Kim and coworkers (2012) could also demonstrate a reduced total wax load in the stems and siliques of the *ltpg1 ltpg2* double-mutant and in the siliques of the *ltpg2* single mutant. No alterations of the total wax load were found for *ltpg1* in this study by Kim et al. (2012).

Overexpression of the *Brassica rapa* BrLTPd1 in *Brassica napus* causes a reduced wax deposition on leaves. When the chemical composition of leaves from a line overexpressing the BrLTPd1 was determined, it was found that the C31 alkane (hentriacontane) was reduced with 78 % and the C29 alkane was reduced with 44 %. Overexpression of BrLTPd1 also induces morphological changes of leaves and flowers in *B. napus* (Liu et al. 2014). There are also several LTPs from monocot plants that have expression patterns suggesting a role in wax or cutin deposition. For instance, the barley HvLTP1.2 (LTP7a2b) has a strong expression in epidermal leaf strips (Hollenbach et al. 1997). The precise role for the LTPs in the cuticular wax synthesis is not clear. The LTPs may act directly in the transport of cuticular lipid through the cell wall or alternatively as a regulatory component for the transport.

### LTPs are functioning in liquid secretion

The tobacco NtLTP1.2 is present in the liquid droplets that are secreted by cells of the long glandular trichomes on the leaves (Choi et al. 2012). In transgenic tobacco that overexpresses NtLTP1.2, there is an increased liquid secretion from the trichomes compared to WT. In plants where NtLTP1.2 expression has been silenced with RNAi, the liquid secretion is decreased. The compounds secreted from the long glandular trichomes confer resistance to insect pests. Consequently, Choi et al. (2012) could show that NtLTP1.2 overexpressing lines have an increased resistance to aphid infestation. The opposite was found for the NtLTP1.2 RNAi silencing lines, which showed increased aphid infestations. Expression in epidermal cells including trichomes was also found for wheat TaLTP1.3. Its promoter is active in young leaves, shoots and spikes but not in roots (Yu et al. 2014). However, no phenotype is yet connected to this wheat LTP.

### LTPs are needed for pollen and seed development

The LlLTP1.1 (SCA) from *Lilium longiflorum* (lily) was the first LTP suggested to have a role in the sexual reproduction of plants (Park et al. 2000). SCA is involved in pollen tube adhesion-mediated guidance during pollen tube growth. It seems that SCA forms an adhesive matrix with pectin that guides the pollen tubes to the ovules (Park et al. 2000). On the basis of sequence similarity, seven SCA-like LTPs were identified in Arabidopsis (Chae et al. 2010). When T-DNA insertion mutants for those seven genes were investigated, only AtLTP1.8 (LTP5) showed a phenotype.

In the *ltp5*-*1* mutant an aberrant, unspliced transcript is accumulating due to the localization of the T-DNA close to 3′ splice recognition site of the only intron in the gene. In the presence of the aberrant *ltp5*-*1* transcript, plants have defects in pollination and seed formation, such as that the majority of the *ltp5*-*1* pollen tubes reach only the middle of the ovary and the *ltp5*-*1* silliques contain significant numbers of unfertilized ovules (Chae et al. 2009). Another T-DNA insertion allele without detectable LTP5 expression does not show any mutant phenotype. Thus, the presence of an aberrant LTP5 in the *ltp5*-*1* mutant seems to contribute to the phenotype as a gain-of-function mutation. Based on the *ltp5*-*1* phenotype, LTP5 is suggested to be involved in establishing or maintaining polar growth of the pollen tube. As revealed from LTP5 promoter:GUS fusion lines, LTP5 has a unusually wide expression pattern with expression in root tips, at initiation sites for lateral roots, hypocotyls, shoot apex, cotyledons, first leaves, pollen, style, and petals (Chae et al. 2010).

After the discovery of SCA1, several other LTPs with a function in pollen development and fertilization have been identified. CaLTPc1 from *Capsicum annuum* L. (chili pepper) was identified as a differentially expressed gene in male fertile lines of chili pepper (Chen et al. 2011). It is strictly expressed during the middle phases of anther development. When virus-induced gene silencing was used to shut down expression, the silenced plants showed normal vegetative growth and flowering. However, the pollen from CaLTPc1-silenced plants had lower germination efficiency and significant shorter pollen tubes. Moreover, a large number of the pollen grains have a defective morphology with deep invaginations.

The rice OsLTPg25 (OsC6) is expressed in tapetal cells and microspores during the post-meiotic stages 9–11 of anther development, according to the developmental stages of the rice flower defined by Zhang and Wilson (2009). In immunological assays, the OsC6 protein was detected in tapetal cell cytoplasm, the extracellular space between the tapetum and the middle layer, as well as in the anther locule and anther cuticle (Zhang et al. 2010). Silencing of OsC6 with RNAi result in reduced pollen fertility. In OsC6 silenced plants, the anthers follow normal development until stage 8. At late stage 9, the development is clearly different in the silenced plants, such as that free young microspores are released from the tetrad. Furthermore, tapetal cells are degenerated and microspores have irregular shapes and became shrunken. At later stages of anther development, the OsC6-RNAi lines develop fewer normal orbicules and irregular pollen walls. Ectopic expression of OsC6 results in granule-like droplets on the inner surface of the tapetal cells. The phenotypes obtained in knock-downs and overexpressors are suggesting a key role for OsC6 in transporting lipophilic material required for proper pollen development from the tapetal cytoplasm to the locule (Zhang et al. 2010).

The Arabidopsis AtLTPc1, AtLTPc2, and AtLTPc3 all have an expression pattern restricted to the tapetum of developing anthers (Huang et al. 2013). When the promoter and coding region of AtLTPc3 was fused to GFP, it was found that during stages 7–9 of floral development (Smyth et al. 1990) AtLTPc3 is localized to the anther locule, while starting from stage 9, it is also associated with the microspore surface. Double RNAi silencing of AtLTPc1 and AtLTPc3 did not reveal any abnormalities on the pollen surface, and the pollen showed no reduction in fertility. However, the intine underneath the exine is somewhat impaired in the stage 11 microspores of RNAi plants, as it appears separated from the exine and the microspore plasma membrane (Huang et al. 2013).

The Arabidopsis LTPgs AtLTPg2, AtLTPg3, AtLTPg4, AtLTPg5, and AtLTPg6 are all involved in the development of pollen and seed. The *Atltpg3*-*1*, *Atltpg4*-*1*, and *Atltpg4*-*2* T-DNA single mutants have deformed or collapsed pollen grains (Edstam and Edqvist 2014). Furthermore, seeds from the single mutants *Atltpg2*-*2*, *Atltpg3*-*1*, *Atltpg4*-*1*, *Atltpg4*-*2*, *Atltpg5*-*1*, *Atltpg6*-*1*, and *Atltpg6*-*2* have an inability to restrict salt uptake. In the case of *Atltpg4*-*1*, *Atltpg4*-*2*, and *Atltpg5*-*1*, the seeds had abnormal phenotypes, such as the protrusion of seed hairs, or a shrunken and deformed appearance. Lipid analysis of the seed coats from *Atltpg4*-*1*, *Atltpg4*-*2*, *Atltpg6*-*1* and *Atltpg6*-*2* revealed a large decrease in ω-hydroxy fatty acids and an increase in unsubstituted fatty acids. Among the unsubstituted fatty acids, the largest difference between the mutant lines and the WT was the increase in C20:0, C22:0, and C24:0 fatty acids in the mutant lines. For the ω-hydroxy fatty acids, the largest decrease is seen for 24-hydroxytetracosanoic acid (C24ωOH), which is an important constituent of suberin. Suberin is one of the main barrier polymers (Vishwanath et al. 2015). It is synthesized to create a hydrophobic barrier against uncontrolled water and solute diffusion through cell walls. Suberin is found in seed coats, but also in perodermal and endodermal cell walls in roots. Seemingly, these Arabidopsis LTPgs are involved in the transport of polyester components to the site of polyester synthesis on the surfaces of pollens and/or seeds.

There are also several examples of LTPs that have an abundant expression in floral organs, fruits or seeds but where a phenotype not yet has been reported. The promoter of rice OsLTPd11 (OsLTP6, OsDIL) is specifically active in anthers from the microspore mother cell developmental stage (stage 6 as defined by Zhang and Wilson 2009) to the mature pollen stage (stage 14) (Guo et al. 2013a; Liu et al. 2013). A pistil-specific LTP, SsLTP1.1, is expressed in the Asteraceae *Senecio squalidus* (Allen et al. 2010). In *N. tabacum*, a pistil-preferential LTP (TOBC065A09) was identified in microarray experiments (Quiapim et al. 2009). A stigma specific LTP (contig Cl000870:1) from *Crocus sativa* L. (saffron) was identified during sequencing of ESTs from a saffron stigma cDNA library (D’Agostino et al. 2007). Microarray analysis and sequencing of a stigma-enriched cDNA library from Arabidopsis revealed that both AtLTP1.5 and AtLTPg21 have enhanced expression levels in the stigma (Swanson et al. 2005). The mung bean VrLTP1.2 (Vrltp1) and VrLTP1.3 (Vrltp2) are both expressed in floral buds and in the embryo during early embryogenesis, but not in mature or dehydrated seeds (Liu and Lin 2003).

### LTPs are important for fruit development and seed germination

Gene expression patterns indicate that LTPs also play important roles during fruit development and seed germination. In *Coffea arabica*, CaLTP2.1, CaLTP2.2, CaLTP2.3, and CaLTP2.4 are expressed in the pericarp and endosperm during fruit development with peaks 90–120 days after flowering (Cotta et al. 2014). The expression of maize ZmLTPd6 (BETL9) is restricted to developing kernels. The transcript could be detected 11 days after pollination and only in RNA extracted from the lower halves of the kernels, thus in the basal endosperm transfer cell (ETC) layer. The closely related ZmLTPd14 (BETL9like) is also specifically expressed in developing maize endosperm within the same time frame as BETL9, but rather in the aleurone cell layer (Royo et al. 2014).

GUS analysis of the promoters of wheat TaLTPd1 (TaPR60) and *Triticum durum* TdLTPd1 (TdPR60) showed that the promoter activity of both genes is restricted to the ETC. The homologous *T. durum* protein TdLTPd2 (TdPR61) has a wider expression pattern in the endosperm, since the promoter is active in the ETC, the aleurone, and the starchy endosperm (Kovalchuk et al. 2009, 2012). In *Euphorbia lagascae*, ElLTP1.1 and ElLTP1.2 are expressed specifically and abundantly in the endosperm during seed germination (Edqvist and Farbos 2002; Eklund and Edqvist 2003).

The expression patterns of Arabidopsis AtLTPd9 (END1) and AtLTPd12 (END2) indicate roles in reproduction also for these proteins (Li et al. 2014b). The AtLTPd9 transcripts are abundant in flowers before pollination and increase even more in young green siliques. Furthermore, when the activity of an AtLTPd9 promoter:GUS fusion was followed in Arabidopsis, particularly strong GUS expression was detected in dividing nuclei, endosperm nodules and in the developing embryo at the globular stage of embryo development. AtLTPd12 transcripts are mainly accumulating in flowers before pollination, and the transcript levels are reduced in the siliques. VuLTP1.1 (VULTP) from *Vigna unguiculata* (cowpea) is another LTP which is accumulating during seed development. VuLTP1.1 is also expressed in seedling leaves, but not in roots, leaves, and flowers of adult plants (Carvalho et al. 2006).

### LTPs are involved in cell expansion

The tobacco NtLTP1.6 (TobLTP2) was demonstrated to have in vitro cell-wall loosening activity (Nieuwland et al. 2005). This is an activity usually attributed to expansins. Pre-incubation of NtLTP1.6 with β-sitosterol or benzene completely abolished the cell-wall loosening activity suggesting that the availability of the hydrophobic cavity is essential for the cell-wall loosening.

### LTPs are important for nodule formation

*Medicago truncatula*, like other legumes, forms N^2^ fixing root nodules after symbiotic interactions with microorganisms. The *M. sativa* MsLTPd1 (MtN5) has a root specific expression pattern, and it is upregulated in response to symbionts, such as *Sinorhizobium meliloti* or pathogenic microorganisms, such as *Fusarium semitectum*. The protein is produced during the early stages of the symbiotic interaction and is localized to mature root nodules (Pii et al. 2012). Ligand-binding studies in vitro showed that MsLTPd1 binds to LLPC and LPPC (Pii et al. 2009). In *M. truncatula* roots where MsLTPd1 expression is silenced with RNAi, there is an increase in root hair curling after rhizobia infection. Furthermore, there is a decrease in the number of invaded roots compared to WT. Nonetheless, the total number of nodule primordia is not varying between WT and MsLTPd1-silenced plants (Pii et al. 2012). From these experiments, Pii et al. (2012) raised the hypothesis that MsLTPd1 is involved in modulating the perception or the activity of rhizobia-derived signal molecules.

Another LTP involved in nodule organogenesis is AsLTP1.1 (AsE246) from *Astragalus sinicus* (Chinese milk vetch) (Lei et al. 2014). Chinese milk vetch can establish a specific endosymbiosis with *Mesorhizobium huakuii* 7653R and form N_2_-fixing root nodules. In lipid binding assays, based on competition with P-96 for binding to AsE246, fatty acids with 16- to 18-carbon chains showed higher competition, while shorter (laurate and myristate) and longer (arachidic acid and behenic acid) fatty acids were competing with less efficiency. Furthermore, also the membrane lipids PC, phosphatidylethanolamine (PE), phosphatidylinositol (PI), digalactosyldiacylglycerol (DGDG), and monogalactosyldiacylglycerol (MGDG) could compete with P-96 for binding to AsLTP1.1. AsLTP1.1 is localized to nodule cells containing symbiosomes. Ectopic overexpression of AsLTP1.1 results in increased numbers of root nodules. When AsLTP1.1 expression is knocked down with RNAi, there is a significant decrease in formed root nodules. The nodules from the RNAi plants also contained fewer infected cells (Lei et al. 2014). It is possible that the function of AsLTP1.1 is related to the transport of plant-synthesized lipids to the symbiosome membrane.

### LTPs could be involved in root suberin synthesis

Other than being important for nodulation, there are likely other functions for LTPs in roots. It has been suggested that LTPs are involved in the synthesis and accumulation of suberin in roots, as also shown for the suberin synthesis in seed coats. The support for a role of LTPs in suberin accumulation in roots is so far based on analysis of expression and co-expression. As described earlier, the LTPgs from Arabidopsis and rice are separated in three expression cluster, of which one of the clusters showed significant co-expression with genes known to be involved in suberin biosynthesis in roots (Edstam et al. 2013). In Arabidopsis, AtLTPg3, AtLTPg4, AtLTPg23, and AtLTPg26 belong to the expression cluster correlating with suberin biosynthesis, while in rice, OsLTPg1, OsLTPg2, and OsLTPg24 were pointed out to have putative roles in suberin biosynthesis. Possibly, the root LTPs could be involved in the trafficking of suberin precursors to polymerization sites in the cell wall. Further experimental studies will be required to determine the precise role of LTPs in suberin synthesis in roots and elsewhere.

### LTPs are involved in defense against biotic stress

There are several studies that suggest that LTPs are toxic for fungal plant pathogens. Several wheat LTPs (TaLTP1.4, TaLTP1.13, TaLTP1.17, TaLTP1.18, TaLTP1.25–1.28) were expressed in *P. pastoris* and then analyzed in in vitro growth inhibition tests (Sun et al. 2008). Most of the tested LTPs showed inhibitory effect for the growth of the wheat pathogens *Puccinia graminis*, *Puccinia triticina*, and *Pyrenophora tritic*-*repenti*. The in vitro toxicity of the LTPs could be derived from an alteration of the fungal membrane permeability, as the fungal uptake of the fluorescent probe SYTOX green increased in the presence of the inhibitory wheat LTPs.

The wheat TaLTP1.14 is associated with resistance against Fusarium head blight, caused by *Fusarium graminearum* as this LTP is 50-fold more abundant in wheat plants carrying the resistant allele Qfhs.ifa-5A (Schweiger et al. 2013). On the other hand, the wheat TaLTP1.23 (Hfr-LTP) shows a 196-fold decrease in abundance in susceptible plants over the first eight days of attack by the virulent Hessian fly larvae. A similar pattern, although with a less dramatic decrease, was also found for TaLTP1.16 (TaLTP3) (Saltzmann et al. 2010). The transcription of Hfr-LTP did not respond to other tested biotic and abiotic stresses. Moreover, the expression of cowpea VuLTP1.1 in seedling leaves is repressed to 60 % after infection with the fungi *Fusarium oxysporum* f. sp. *phaseolus* (Carvalho et al. 2006).

### LTPs are involved in defense against abiotic stress

The LTPs are often reported to be important for tolerance to abiotic and biotic stresses in plants. Still, there are, to our knowledge, no examples of a plant where either knock-out, knock-down or overexpression of an LTP result in a phenotype showing increased sensitivity or increased tolerance to stress. Anyway, there are many cases where the expression of LTP-genes is responding to abiotic stresses like drought, cold, and salt or to phytohormones, such as abscisic acid (ABA). Here, we will only give some examples from recently published reports. For instance, the transcript levels of Arabidopsis AtLTP1.12 are dramatically induced by dehydration and ABA treatment (Guo et al. 2013b). LjLTP1.1 (LjLTP6) and LjLTP1.3 (LjLTP10) from Lotus japonica are specifically expressed in aerial tissues. Both genes are highly induced during drought (Tapia et al. 2013). The expression of rice OsLTPd11 is also greatly induced by drought and also by PEG, NaCl, cold, and ABA (Guo et al. 2013a). The wheat LTPs TaLTP1.2 and TaLTP1.13 are upregulated during drought, chilling stress, and wounding (Yu et al. 2014). In maize, 14 LTPs are differentially regulated by drought, salt and/or re-watering treatments, while three other maize LTPs are upregulated during cold stress (Wei and Zhong 2014).

When the expression of eight LTPgs in the moss *P. patens* was investigated during different stress treatments, cold and dehydration caused a significant upregulation of several of the genes. For instance, PpLTPg3, PpLTPg8, and PpLTPg9 are significantly upregulated after cold treatment, while PpLTPg5 is downregulated. Dehydration causes a significant upregulation of PpLTPg2, PpLTPg3, PpLTPg6, and PpLTPg9. Thus, PpLTPG3 and PpLTPG9 are upregulated after both cold treatment and dehydration. Treatment of the moss with UV-B radiation, ABA, and salt leads to downregulations of the PpLTPg genes (Edstam et al. 2014).

## Summary and outlook

Plants conquered land at least 500 million years ago. Since those days the LTPs have carried out necessary, life-supporting functions in all land plants, in all tissues and during all stages of the life cycle. In this review, we have summarized the current information about the 3D structure, ligand binding, gene expression, and phenotypic investigations regarding the LTPs. The first 3D structures of LTPs were presented in the early 1990s, and a quite large number of papers describing the structural or ligand binding properties of LTPs have been published since then. The hydrophobic ligand-binding cavity is flexible and often swells when a lipid ligand is binding. Several LTPs fit two fatty acyl chains in their binding cavities. Upon binding a ligand, the structural alterations are quite limited and significant structural rearrangements occur mainly in the C-terminal part of the LTPs. In other parts of the protein, there may be more local rearrangements of 1–2 amino acids. A Tyr residue close to the opening of the cavity, Tyr79 in TaLTP1.1, is a key residue in many LTPs. Seemingly, the orientation of this Tyr residue often controls the shape, size, and binding capacity of the hydrophobic cavity. In unliganded LTP structures, Tyr79 often collapsed into the ligand binding cavity. Many ligands cause a shift in the orientation that move the aromatic ring of Tyr79 outwards to the solvent. This orientation excludes the formation of hydrogen bonds between Tyr79 and the ligand. Tyr79 may act as a gate keeper of the cavity and the rotation ensures that high specificity interactions with ligands are avoided. However, Tyr79 in TaLTP1.1 has an important role in the binding of PGB_2_ as in that case rotation of Tyr79 enables a hydrogen bond between the carboxyl group of the ligand and the hydroxyl group of Tyr79. It is clear that the properties of the ligand influence the 3D structure of the LTP.

Several in vitro binding experiments show that LTPs bind both saturated and unsaturated fatty acyl chains, presented in various molecules, such as in LPC, PG, acyl-CoA or as free fatty acids. Some LTPs are reported to bind to hydroxylated acyl chains, while only rice LTP2 is reported to bind to a sterol. The dissociation constants (*K*_d_) for LTP-ligand interactions are commonly in the micromolar range indicating that the LTPs are involved in low-affinity interactions. The preferred ligands are, in most cases, fatty acyl chains with 14–18 carbons. Unfortunately, the ligand-binding studies have rarely improved our functional understanding of the LTPs. We are still rather clueless about their in vivo binding repertoire. However, we probably have to accept that any lipid or other hydrophobic molecule, within a certain molecular size range, will fit into the cavity of these promiscuous proteins.

LTPs are expressed in all tissues and at every developmental stage of the plant. Nevertheless, each unique LTP is likely acting in a very specific set of tissues during specific stages of the life cycle. Based on the gene expression patterns, the LTPs in seed plants can be functionally classified into root LTPs, green LTPs, and reproductive LTPs, expressed in roots, green tissues, and floral tissues, respectively. This functional classification fits well with the experimental evidence showing that the LTPs are involved in the synthesis of cuticular waxes on leaves, in suberin synthesis in seed coat and roots, in sporopollenin synthesis of the exine walls of pollen grains, in adhesive polymer synthesis in the style (Park et al. 2000). Clearly, the major role of many LTPs is in the accumulation of the complex barrier polymers on the surfaces of tissues and organs in plants. These lipid-based polyesters form barriers that control the fluxes of gases, water, and solutes, and also play roles in protecting plants from biotic and abiotic stresses and in controlling plant morphology and reproduction. The evolution of the biosynthesis of these polymers was absolutely essential for the successful colonization of land by plants for approximately 500 million years ago (Wellman et al. 2003).

The cuticle, suberin, and sporopollenin polymers all have an extremely complex and heterogeneous nature, and the details of their synthesis are still elusive. The lipid polymer synthesis requires the de novo synthesis of polymer precursors, the massive secretion and export from the lipid bilayer. Once exported from the plasma membrane, the extremely hydrophobic polymer compounds have to pass through an outer compartment, such as the apoplast or the locule, to the actual polymerization sites. The data we have reviewed here suggest that many LTPs are important for this cell exterior transport of precursors for the synthesis of lipid-based polymers.

**Fig. 9:**
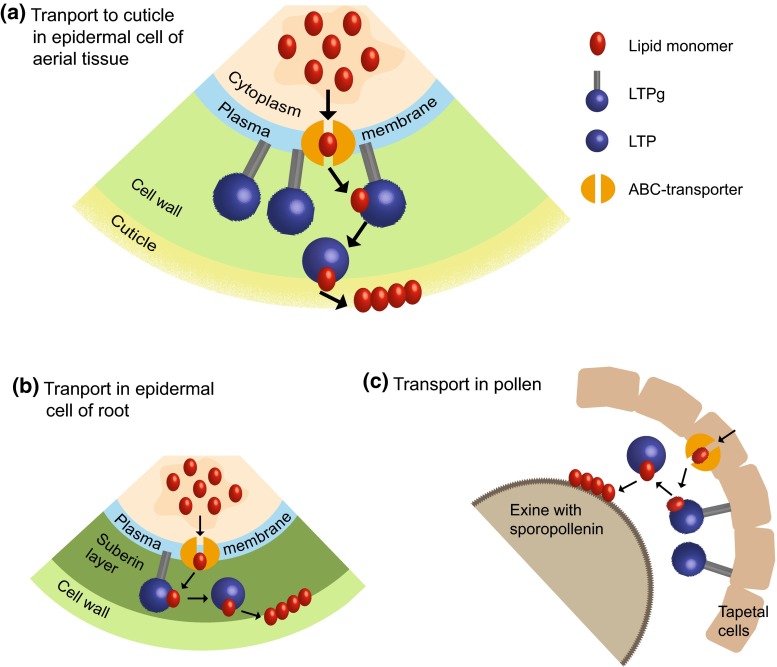
A schematic model describing the proposed functions for LTPs in green tissues (**a**), in roots (**b**), and in pollen (**c**)

It is still rather unclear how the LTPs are running the exterior transport of building blocks for lipid polymer synthesis. There are some low-resolution models, which attempt to explain the rational and mechanisms behind the role of LTPs in polymer synthesis. In these models, ABC transporters are moving lipid polymer components through the plasma membrane. On the exterior side of the plasma membrane, the lipids are transferred to LTPs. The LTPs continue the transport and shuttle the lipid polymer components from the plasma membrane to the sites of polymer synthesis, which, for instance, could be the surfaces of stems or pollen (Fig. 9). Possibly, the ABC transporters deliver the polymer building blocks to LTPgs, which are attached to the membrane through their GPI-anchor. The cargo is then transferred from LTPg to other LTPs that may diffuse freely in the space outside the plasma membrane (Fig. 9). The challenge now is to design experiments that could approve or disapprove this model for LTP function. For this purpose, it will, for instance, be useful to obtain conclusive results regarding the in vivo localization and the in vivo molecular interactions of individual LTPs. One way forward could be to use sophisticated visualization tools, such as super-resolution microscopy, to follow the movement of specific LTPs in the plant.

Based on our current knowledge, the major LTP types are not functionally specialized. Rather, LTPs from one single type are often involved in many different processes in several parts of the plants. LTP1 are involved in cell expansion, lipid secretion, nodule development, and pollen tube growth. LTPd is important for systemic resistance signaling, cuticular wax accumulation and are also involved in nodule development. The GPI-anchored LTPg are also active in a number of different processes, such as cuticular wax accumulation, pollen exine formation, and seed coat suberin polymerization. LTPc seems to be more functionally specialized. The LTPc genes are strictly expressed in developing anthers where the LTPc proteins probably are involved in the transport of lipid required for the biosynthesis of the pollen exine.

One would expect that there are identifiable features in sequence or structure separating LTPs involved in the synthesis of one lipid polymer from the LTPs involved in synthesis of another polymer. Thus, sequential or structural motifs would reveal whether the LTP is involved in, for instance, cutin biosynthesis in stems and leaves or required for the suberin biosynthesis in seed coats. There could, for example, be similarities in the ligand binding cavity or on the protein surface. So far, such distinguishable features have not been identified. However, one should note that both the functional and structural investigations are in a rather early stage. Most of the structural studies have been done on LTPs from seeds, and there are just a few protein structures from LTPs expressed in other tissues. Furthermore, the mechanistic details of how the LTPs act are still lacking. The accumulation of more data may reveal that LTPs involved in the same or similar process share structural characteristics important for their specific functional purpose.

Interestingly, there are some LTPs, which are involved in other processes than lipid polymer biosynthesis. One example is AtLTPd1 (DIR1), which is involved in SAR following the exposure to a pathogen. During SAR, DIR1 is moving from the induced leaf down the petiole to distant leaf petioles (Champigny et al. 2013). The highly similar protein AtLTPd2 (DIR1-like) may also contribute to SAR, as the *dir1*-*1* mutant displays a partially SAR-competent phenotype (Champigny et al. 2013). G3P is one of several chemical inducers of SAR (Gao et al. 2014). DIR1 and also the LTP-like AZI1 are reported to be required for pathogen-induced biosynthesis of G3P in Arabidopsis (Yu et al. 2013). The detailed mechanisms how DIR1 activates SAR have not been revealed yet. At present, there is no experimental evidence suggesting similar roles for other LTPs. Anyway, it would be rather peculiar if only one or two proteins of the large LTP family would be involved in signaling, it is tempting to speculate that we will soon learn about other LTPs with its main function in signal transduction.

The research on LTP has now formed a solid ground, and it is a good time to advance forward with more exciting experiments on these intriguing and fascinating plant proteins. There are many challenges involved in LTP research, such as the intricate gene families resulting in gene redundancy, the low specificity of the ligand:LTP interactions and the complex nature of the lipid polymer synthesis. Anyway, with systematic approaches, it will definitely be possible to significantly advance our knowledge in a few years from now. The reward may be a much improved knowledge about the details of LTPs, but also more importantly an advanced general understanding about plant evolution and physiology as well as about protein function and structure. One way forward could be to use sophisticated microscopy to trace the movements of LTPs in living cells. However, it is also of importance to continue with more basic experiments such as knock-down or overexpression strategies followed with detailed phenotypic investigations. So far, crystal and solution 3D protein structures have mainly been obtained from seed LTPs. Therefore, it is very important to get protein structure information for other LTPs expressed in other tissues and organs. There could also be more emphasis on finding in vivo interaction partners, such as lipids or proteins. Furthermore, the LTPs have several properties, such as the promiscuous lipid binding, flexible binding cavity, and the extreme thermostability, which could open up for valuable applications in the development of biosensors and nanomaterials (Pagano et al. 2013). We expect that the next few years will be a very productive and exciting period for the LTP research.
